# Computer vision-based steering path visualization of headlands in soybean fields

**DOI:** 10.3389/fpls.2025.1673567

**Published:** 2025-10-08

**Authors:** Yuyang Ren, Bo Zhang, Yang Li, Changhai Chen, Wenxiao Li, Yongcai Ma

**Affiliations:** ^1^ College of Information and Electrical Engineering, Heilongjiang Bayi Agricultural University, Daqing, Heilongjiang, China; ^2^ Science and Technology on Special System Simulation Laboratory, Beijing Simulation Center, Beijing, China; ^3^ Agricultural Mechanization Research Division, Harbin Academy of Agricultural Sciences, Harbin, Heilongjiang, China; ^4^ College of Engineering, Heilongjiang Bayi Agricultural University, Daqing, Heilongjiang, China

**Keywords:** YOLO-PFL model, 3D localization, feature detection, crop row centerline extraction, navigation line visualization

## Abstract

**Introduction:**

To address the insufficient accuracy of autonomous steering in soybean headland areas, this study proposes a dynamic navigation line visualization method based on deep learning and feature detection fusion, enhancing path planning capability for autopilot systems during the soybean V3–V8 stage.

**Methods:**

First, the improved lightweight YOLO-PFL model was used for efficient headland detection (precision, 94.100%; recall, 92.700%; mAP@0.5, 95.600%), with 1.974 M parameters and 4.816 GFLOPs, meeting embedded deployment requirements for agricultural machines. A 3D positioning model was built using binocular stereo vision; distance error was controlled within 2.000%, 4.000%, and 6.000% for ranges of 0.000–3.000 m, 3.000–7.000 m, and 7.000–10.000 m, respectively. Second, interference-resistant crop row centerlines (average orientation angle error, –0.473°, indicating a small systematic leftward bias; mean absolute error, 3.309°) were obtained by enhancing contours through HSV color space conversion and morphological operations, followed by fitting feature points extracted from ROIs and the crop row intersection area using the least squares method. This approach solved centerline offset issues caused by straws, weeds, changes in illumination, and the presence of holes or sticking areas. Finally, 3D positioning and orientation parameters were fused to generate circular arc paths in the world coordinate system, which were dynamically projected across the coordinate system to visualize navigation lines on the image plane.

**Results and discussion:**

Experiments demonstrated that the method generates real-time steering paths with acceptable errors, providing a navigation reference for automatic wheeled machines in soybean fields and technical support for the advancement of intelligent precision agriculture equipment.

## 1 Introduction

Intelligent agriculture is an important direction for future agricultural development, but to achieve this goal it must rely on the informatization and intelligence of agricultural machinery. With the continuous advancement of this process, the application of automatic navigation of agricultural machinery in precision agriculture has become increasingly widespread. Current methods of automatic navigation technology can be divided into three categories: global navigation satellite system (GNSS) (Huang et al., 2022), LiDAR (Pin et al., 2025; Chen et al., 2025), and computer vision (CV) (Wang et al., 2023). During field operations, especially in headland areas, buildings, trees, and other obstacles often cause signal interference, resulting in inaccurate positioning. Therefore, it is difficult for GNSS to meet the needs of steering into ground operations to obtain real-time information about the surrounding environment and to avoid damaging crop rows. In contrast, lower-cost CV technology can interpret and perceive the surrounding road environment, static and dynamic objects, and crop growth scenes, compensating for the shortcomings of GNSS absolute positioning methods and the limitations of LiDAR application scenarios.

In recent years, CV-based automatic navigation technology has received increasing attention from researchers. Environmental perception is an important foundation and primary step toward achieving safer, more accurate, and more efficient autonomous driving and navigation systems for agricultural machinery. Environmental perception techniques can be categorized into feature detection algorithms and machine learning algorithms. Feature detection–based algorithms use color (Li X et al., 2022; Xu et al., 2023), texture (Zheng et al., 2025), edges (Zhang et al., 2022), and other features in images to identify possible driving areas. However, machine learning–based algorithms adapt better to environmental changes. These include traditional machine learning (Li C et al., 2024; Zhang et al., 2020) and deep learning (Wang et al., 2024; Lin et al., 2025). By constructing and training parametric models, these methods can automatically learn environmental features and accomplish the task of target detection.

According to imaging principles, vision sensors are divided into 2D vision imaging sensors and 3D stereo vision imaging sensors. Depending on environmental conditions and task requirements, it is necessary to choose a suitable vision sensor. Common vision sensors include monocular sensors, stereoscopic sensors, and RGB-D sensors. Color detection and edge detection are commonly used in monocular vision systems to identify objects and obstacles in agricultural environments due to their low computational effort, mature algorithms, and simple structures, and they are widely applied in agricultural navigation robots. However, monocular vision loses depth information and cannot accurately determine object size, position, and distance (Bai et al., 2023). RGB-D cameras are inevitably disturbed by sunlight, multipath effects, and motion artifacts, making them unsuitable for outdoor applications (Li J et al., 2022). In contrast, 3D images are reconstructed by stereo disparity, which is relatively insensitive to changes in lighting in agricultural environments. Stereo vision also uses geometric constraints to calibrate the ground when cameras are fixed on uneven terrain, and it has good range detection capabilities in addition to color and feature detection, thereby enhancing target detection in agricultural environments (Ding et al., 2022).

Accurate positioning of navigation targets is a prerequisite for achieving automatic navigation. In complex field environments, target recognition is affected by factors such as crop occlusion and light changes, which pose challenges to accurate positioning. Agricultural machines must accurately recognize targets in irregular field conditions to ensure they can perform their tasks effectively. Zheng et al. (2023) proposed a vision system based on an improved lightweight YOLOX-Nano architecture combined with a K-means clustering algorithm for accurate autonomous navigation path detection in a jujube catch-and-shake harvesting robot. This method effectively addressed the challenge of extracting navigation paths in complex orchard environments with numerous interfering factors. The improved YOLOX-Nano model achieved an mAP of 84.08% with a model size of only 12.30 MB, meeting the requirements for embedded deployment. Wu et al. (2025) proposed an improved DRAM-DeepLabv3+ model for accurate boundary detection in agricultural headland regions by integrating depth information and an enhanced attention mechanism. This approach improved the mIoU by 2.4%, while reducing computational complexity to 27.9 G, decreasing the number of parameters to 19.7 M, and achieving an inference speed of 23.1 FPS. Li et al. (2023) constructed a deep learning–based network combining a convolutional neural network (CNN) and a recurrent neural network (RNN) for headland semantic segmentation. Image preprocessing techniques and a distance-based boundary point clustering algorithm were applied to the headland segmentation mask to obtain the boundary line on the working side of agricultural machinery. This method achieved an mIoU of up to 95.7%. The mean deviation of boundary line extraction on 256*144 resolution images was 3.57 pixels, and the detection rate reached 25.8 FPS. He et al. (2022) proposed MobileV2-UNet to accurately segment farmland areas, achieving an mIoU of 0.908. Frame correlation and RANSAC were applied to detect side and end boundaries. Angular and vertical errors for boundary line detection were 0.865° and 0.021, respectively. Hong et al. (2022), addressing the limitations of GNSS navigation in field turns, proposed a binocular vision–based field ridge boundary recognition and ranging method. By combining Census transform, multiscale segmentation trees, and other optimization strategies, they improved the quality of parallax maps and boundary detection accuracy. Experimental results showed that the method achieved a recognition rate of 99% in the 5–10 m range, with a ranging error as low as 0.075 m. It demonstrated real-time capability on the Jetson TX2 platform, making it suitable for early operations in farmland environments. Luo et al. (2022) proposed a fast and robust multi-crop harvesting edge detection method based on stereo vision, combined with dynamic HSV spatial ROI extraction and coordinate transformation. Field tests showed that the detection accuracy of this method was over 94% in rice, oilseed rape, and maize. Although there are many methods for target detection, most rely on large models that are not favorable for porting to embedded or mobile devices. Therefore, lightweight target detection methods have been developed to improve suitability for deployment in resource-constrained agricultural smart equipment, providing a feasible solution for real-time target recognition and path planning in complex field environments.

On the basis of target positioning, extracting the centerline of the crop row is the key step to align with the ridge.

Yang et al. (2023) proposed a crop row detection algorithm based on autonomous ROI extraction. A YOLO network predicted the agricultural machinery’s traveling area end-to-end. The prediction boxes were unified into an ROI, and the crop and soil background were segmented within the ROI using the Excess Green operator and Otsu’s method. Next, crop feature points were extracted by the FAST corner detection technique, and crop row lines were fitted using the least squares method, achieving an average error angle of 1.88°.

Guo et al. (2024) utilized a model built on the Transformer architecture to learn the elongated structures and global context of crop rows, achieving end-to-end output of crop row shape parameters. Experiments demonstrated that the adopted CRPP architecture provided a solid framework for predicting crop row parameters, with only 0.7 M parameters.

Diao et al. (2023) proposed a centerline detection algorithm based on ASPP-UNet to address the difficulty of identifying the centerline of maize crop rows in complex farmland environments. By combining an improved vertical projection with the least squares method, this approach significantly improved precision and efficiency, achieving an accuracy rate of 92.59%.

Ma et al. (2021) proposed a rice root row detection algorithm based on linear clustering and supervised learning to determine the number of crop rows through dynamic threshold segmentation of the vegetation index combined with horizontal stripe clustering. Parametric regression equations of crop rows and root rows were then established using linear clustering to eliminate edge interference. Experiments showed detection accuracies of 96.79%, 90.82%, and 84.15% at 6/20/35 days after transplanting, respectively.

Zhang et al. (2023) proposed a feature engineering–based crop row recognition method that combined RGB and depth images, with feature dimensionality reduction and an SVM model for crop row centerline detection. The experimental results showed that the best detection accuracy and robustness were achieved when using a radial basis function (RBF) kernel classifier, with a heading angle deviation of 0.80° and a lateral position deviation of 0.90 pixels.

Zhai et al. (2022) proposed a crop row recognition and tracking method based on binocular vision and an adaptive Kalman filter. The method performed image segmentation through the super green–super red model and maximum interclass variance method, extracted vegetation features, detected the centerline of the crop rows using PCA-based detection, and applied a Kalman filter for tracking. Experimental results showed that the recognition rate was 92.36% and the heading deviation was 0.31°.

Li S et al. (2024) collected high-resolution field images by UAV and improved the YOLOv5s model to enhance the detection accuracy of small targets and occluded seedlings. A weighting strategy was introduced to address sample imbalance. Crop row identification was achieved through a slice-assisted inference framework and an improved DBSCAN clustering algorithm, resulting in a clustering accuracy of 100% and a centerline fitting angular error of 0.2455°.

Despite improvements in existing methods, recognition accuracy remains limited in complex field environments, such as those with changing light or complex soil backgrounds. Therefore, optimizing algorithms to improve accuracy continues to be a key challenge.

This study focuses on the visualization of circular arc steering paths, aiming to construct a high-precision visual navigation system using CV and image processing techniques to enhance the automatic steering ability of wheeled agricultural machines in soybean headland areas during the V3–V8 stage (from the full expansion of the third trifoliolate leaf to the full expansion of the eighth trifoliolate leaf). The overall algorithmic process includes three core components:

- Target detection of RGB images of soybean headlands acquired by OAK-D-S2 visual sensors through the YOLO-PFL model, combined with binocular stereo vision to obtain 3D coordinates of crop ridges.
- Extraction of crop row contours within the ridge based on color space conversion and image morphological operations, followed by estimation of the relative azimuth angle between the crop rows and the camera through feature point extraction and least squares fitting of the centerline.
- Determination of the circular arc steering path in the world coordinate system by combining the position of the headland and the direction of the crop row, and projection of this path back to the image coordinate system to visualize it on the image.

## 2 Materials and methods

### 2.1 Local path steering visualization algorithm

This study used PyCharm software for algorithm development, combined with computer vision technology, to complete the arc steering path visualization study. The overall algorithmic process was divided into three main parts: crop row headland positioning, estimation of the relative azimuth angle between crop rows in the ridge and the camera, and generation of the arc turning path in the world coordinate system with visualization.

The first step was to annotate RGB images of soybean headlands collected by image sensors and to divide the dataset. Target detection was implemented using the YOLO-PFL model, and 3D coordinates of the target were calculated using the depth information of each pixel point in the images acquired by binocular stereo vision technology and the camera’s internal parameters.

In the second step, crop row contours were extracted by color space conversion and morphological operations. Regions of interest (ROIs) were defined to extract feature points. The relative angle of the centerline was calculated after the centerline was obtained by fitting the feature points using the least squares method.

**Figure 1:**
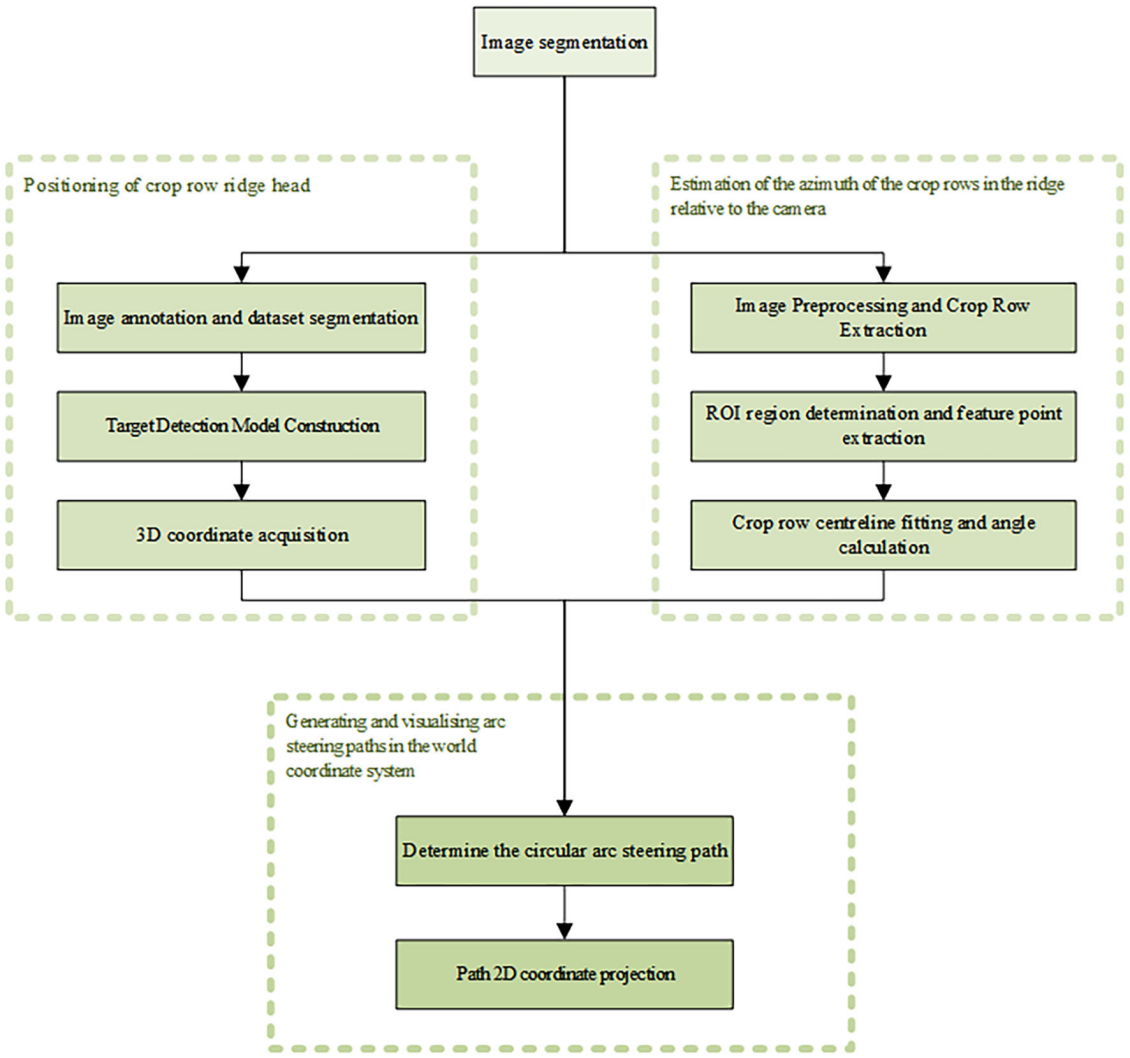
The local path-orientated visualization process proposed in this paper.

The third step determined the circular arc steering trajectory based on the relative 3D coordinates of the headland and the camera obtained in the first two steps, and on the relative angle between the crop rows in the ridge and the direction of advance of the wheeled agricultural machine. The trajectory was then projected back into the image coordinate system to achieve visual display of the steering path. The complete process is shown in **Figure 1**.

### 2.2 Image data acquisition

**Figure 2:**
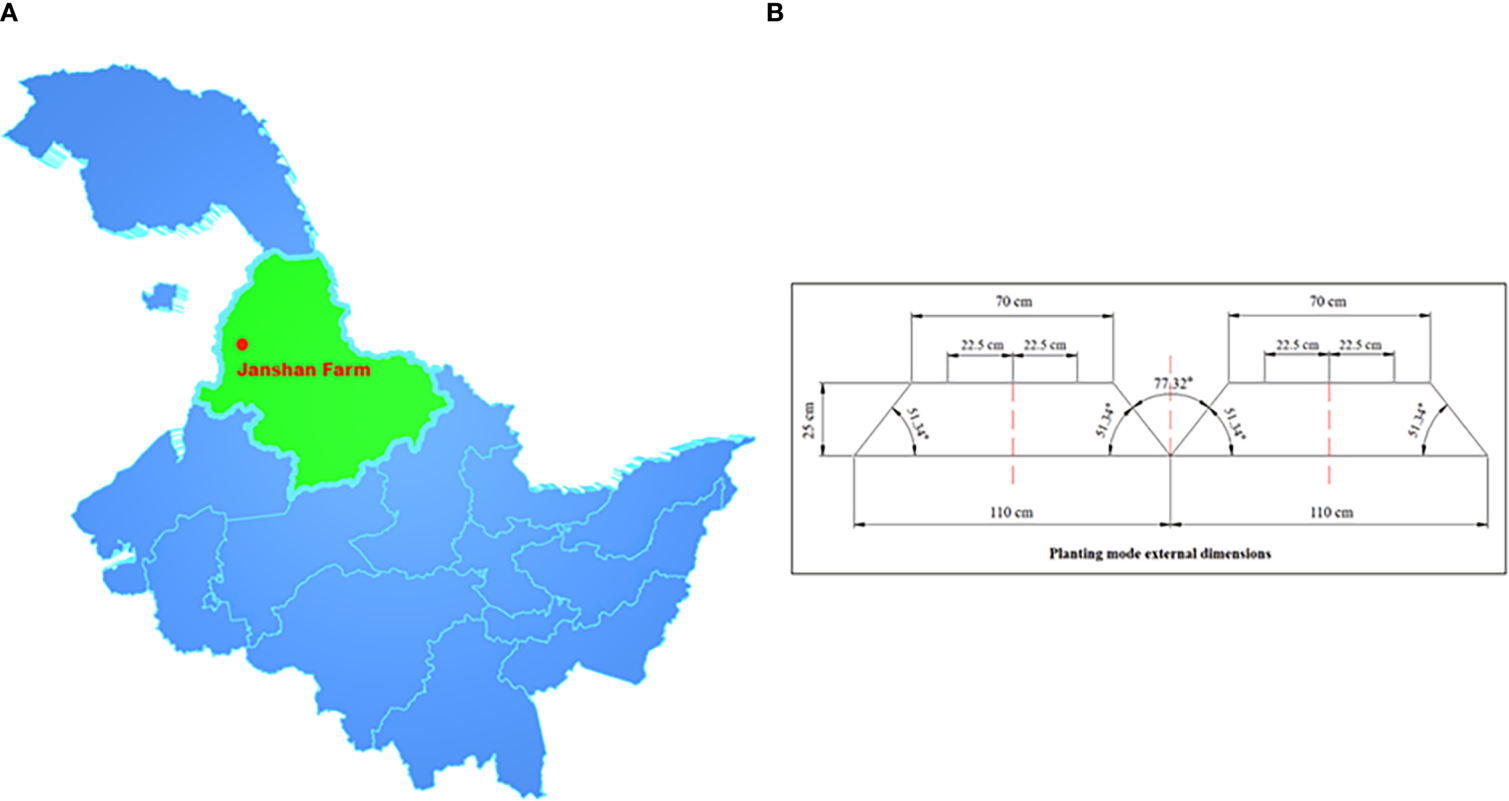
Experimental locations and planting patterns. **(A)** Location of Jianshan Farm, Heihe City, Heilongjiang Province, China. **(B)** Three-row planting pattern on soybean ridges.

In this study, soybean during the V3–V8 growth stages was used as the research object. The location was Jianshan Farm Science and Technology Park, Heihe City, Heilongjiang Province, China (48°52′17.16″N, 125°25′9.40″E), as shown in **Figure 2A**. The soybean planting pattern in this area was three rows on a ridge, with a bottom width of 1.100 m, a platform width of 0.700 m, a height of 0.250 m, a 77.32° angle between the two ridges, and a 51.34° angle of inclination of the platform. Three rows of soybeans were planted on the centerline of the ridge platform, offset to the left and right by 0.225 m, respectively, as shown in **Figure 2B**.

**Figure 3:**
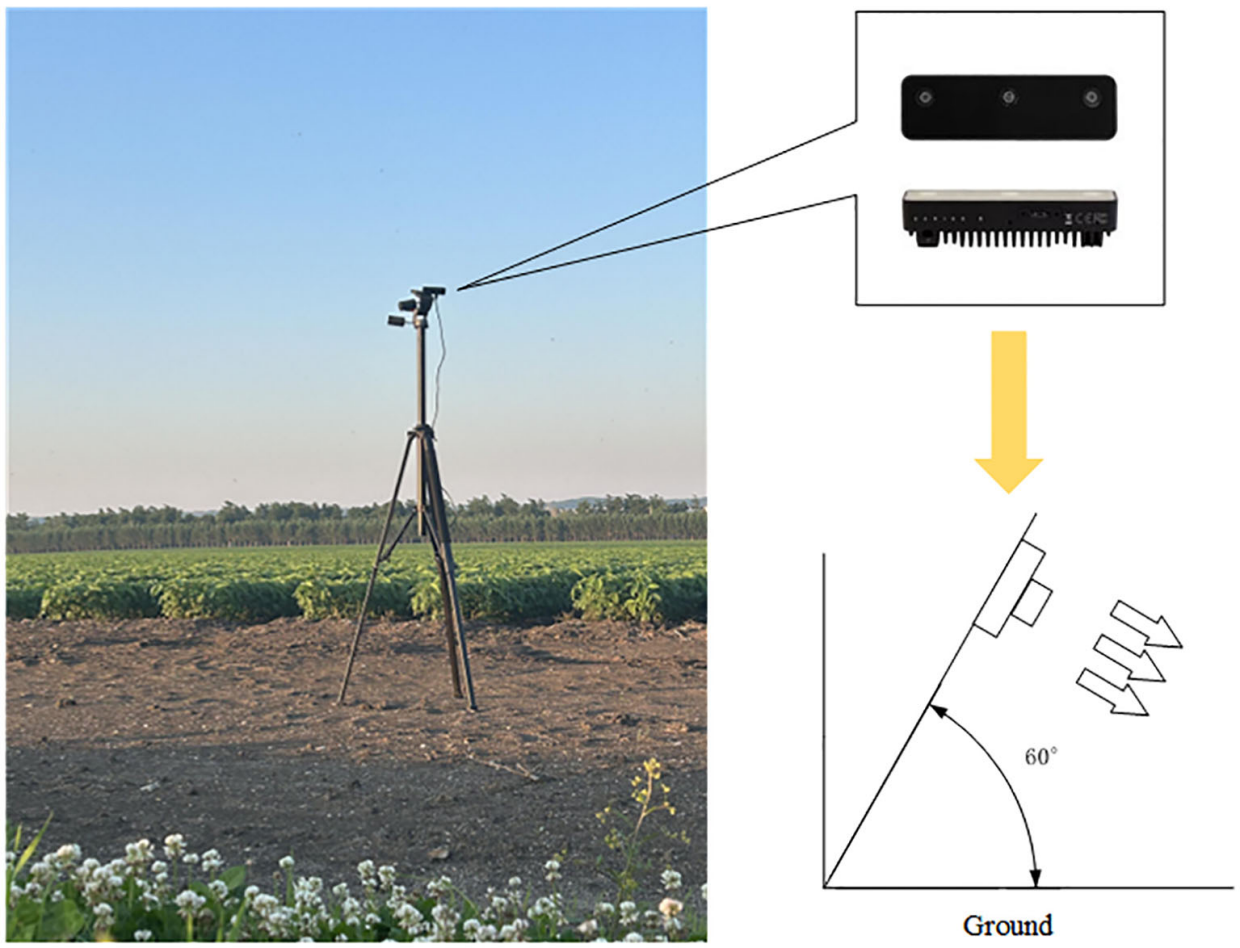
Image sensor placement and shooting angle.

The image acquisition equipment included two OAK-D-S2 vision sensors with three lenses: an RGB camera (monocular color camera) and a binocular camera (stereo vision system consisting of two black-and-white cameras). The OAK-D-S2 was mounted on a camera support 2 m above the ground at an angle of 60.000° to the ground. One image sensor was oriented in the forward direction of the wheeled agricultural machine for headland positioning, while the other was oriented perpendicular to the forward direction, toward the soybean crop, for estimation of the azimuth angle of crop rows in the ridge relative to the camera. The placement and shooting angle of the image sensors are shown in **Figure 3**.

To simulate the movement of agricultural machinery in the field, this study performed OAK-D-S2 displacement operations and simultaneously captured image data of soybeans during the V3–V8 growth stages using both RGB and stereo cameras, under sunny and cloudy conditions, to ensure the model adapted to real field environments. All videos were saved in MP4 format and read frame by frame using OpenCV. Frames were selected based on set intervals or key frame detection methods for subsequent analysis and processing.

### 2.3 Positioning of crop row headland

#### 2.3.1 Annotation and datasets segmentation

**Figure 4:**
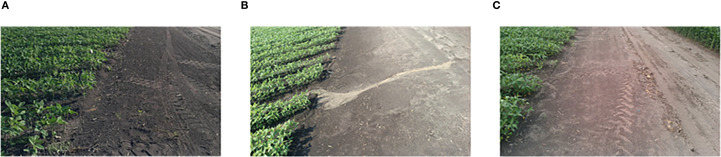
Sample images from the dataset. **(A)** Sunny, V3-V4 period. **(B)** Sunny, period V5-V6. **(C)** Cloudy, period V7-V8.

A total of 1,800 images were selected, some of which were cropped. Of these, 600 were from each of the V3–V4, V5–V6, and V7–V8 stages. The LabelImg software was used to annotate the selected images. The annotated rectangular boxes generated corresponding TXT files containing the category as well as the center coordinates, length, and width information of the boxes. The datasets included a category named “headland.” To ensure uniformity, the datasets were randomly divided into 1,260 images for the training set, 180 images for the validation set, and 360 images for the testing set in a 7:1:2 ratio (**Table 1**). Each dataset contained both image and label information, and sample images are shown in **Figure 4**.

**Table 1:** Distribution table of dataset.

Categories	Number of images
Training set	1,260
Validation set	180
Testing set	360

#### 2.3.2 Target detection model construction

**Figure 5:**
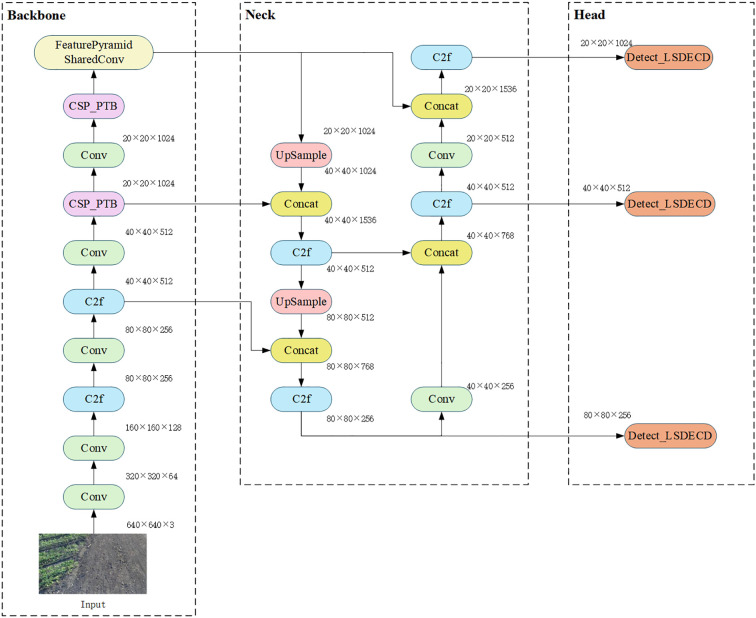
YOLO-PFL network structure diagram.

An improved target detection algorithm, YOLO-PFL, was proposed in this study, and its network structure is shown in **Figure 5**. The model was based on YOLOv8, and its structure introduced a partial Transformer modeling mechanism and a fine-grained feature fusion strategy while maintaining the advantages of the original YOLO framework, in order to improve target detection capability in multi-scale and complex scenes. Its main improvements were reflected in three aspects: the introduction of the CSP-PTB (Cross Stage Partial-Partially Transformer Block) module to replace the partially convolutional structure in the backbone network in order to enhance the global modelling capability; the inclusion of the FeaturePyramidSharedConv module in the feature fusion stage that used shared convolutional kernels to achieve multi-scale context awareness; and introducing LSDECD (Lightweight Shared Detail Enhanced Convolutional Detection Head) (Sun et al., 2025), a lightweight detail enhancement module, in the detection head part to enhance the detection accuracy and efficiency.

**Figure 6:**
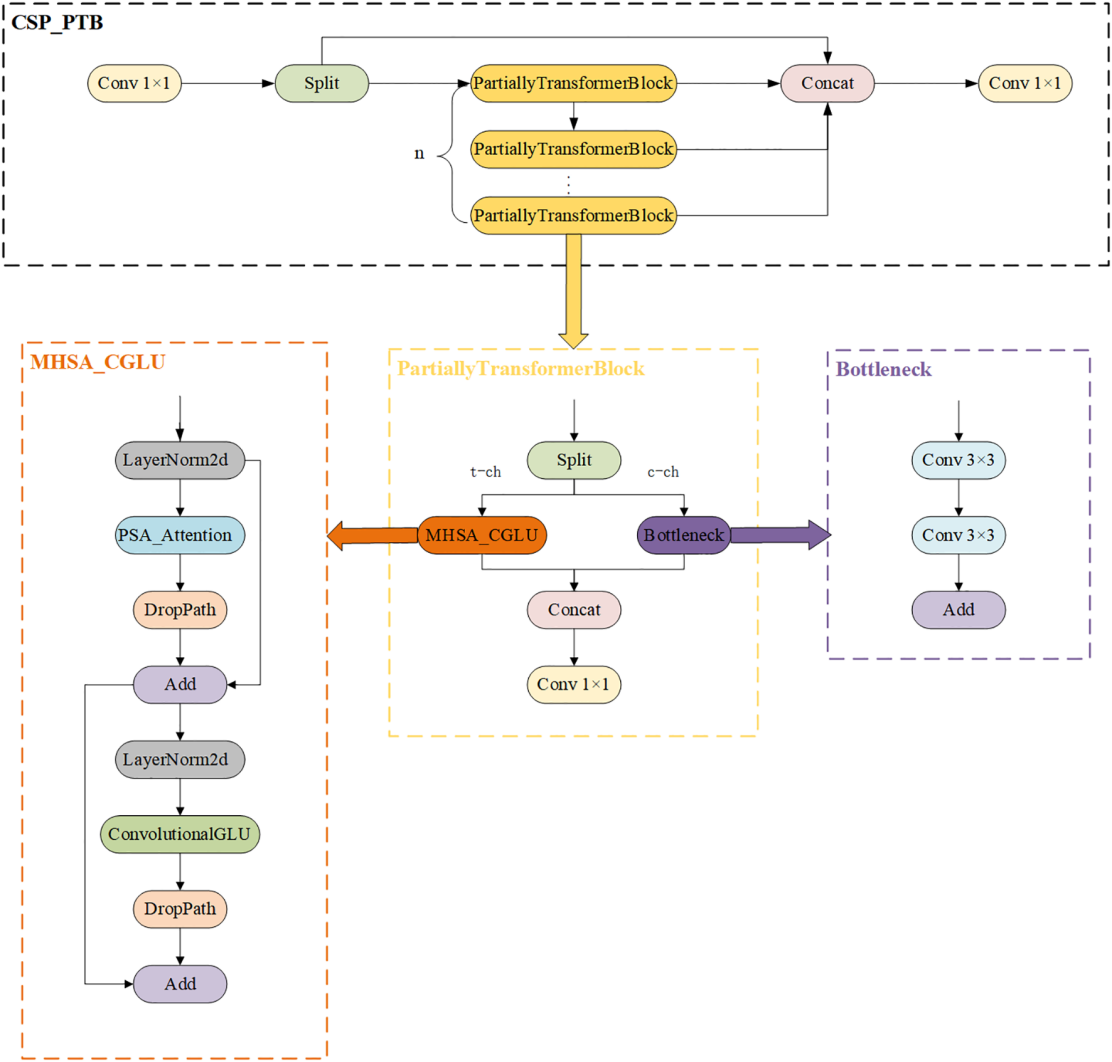
CSP_PTB structure diagram.

**Figure 7:**
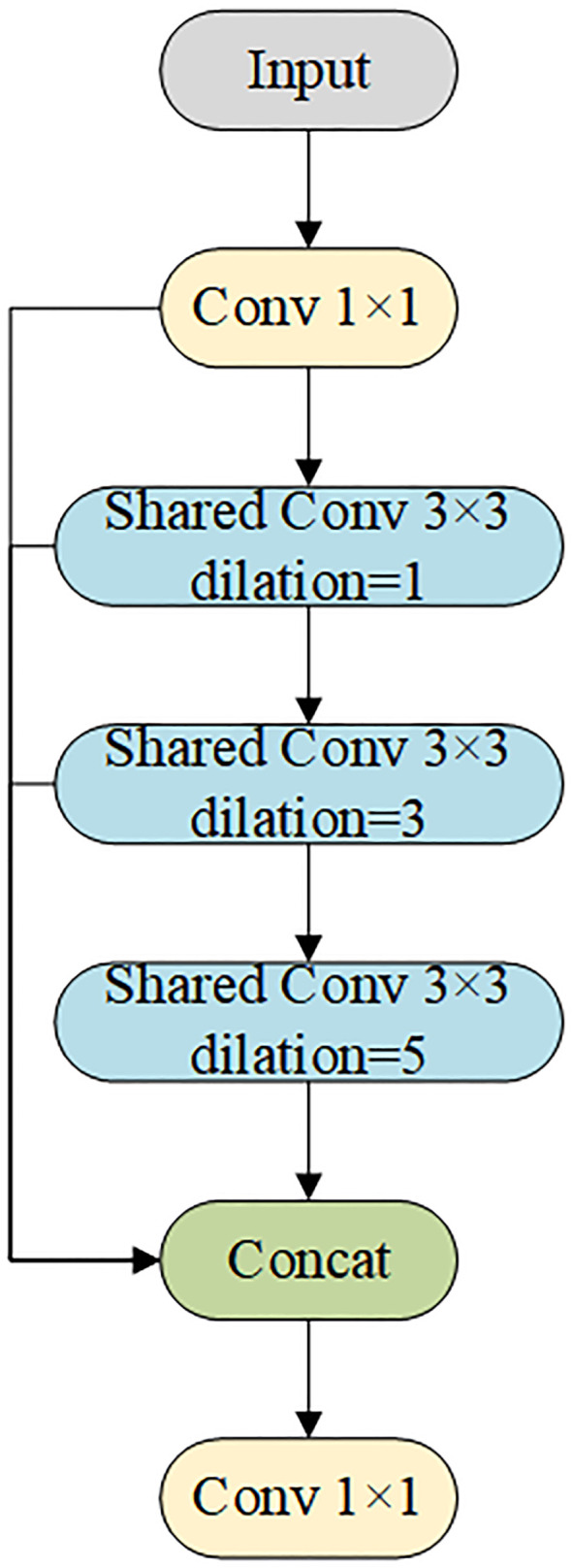
FeaturePyramidSharedConv structure diagram.

**Figure 8:**
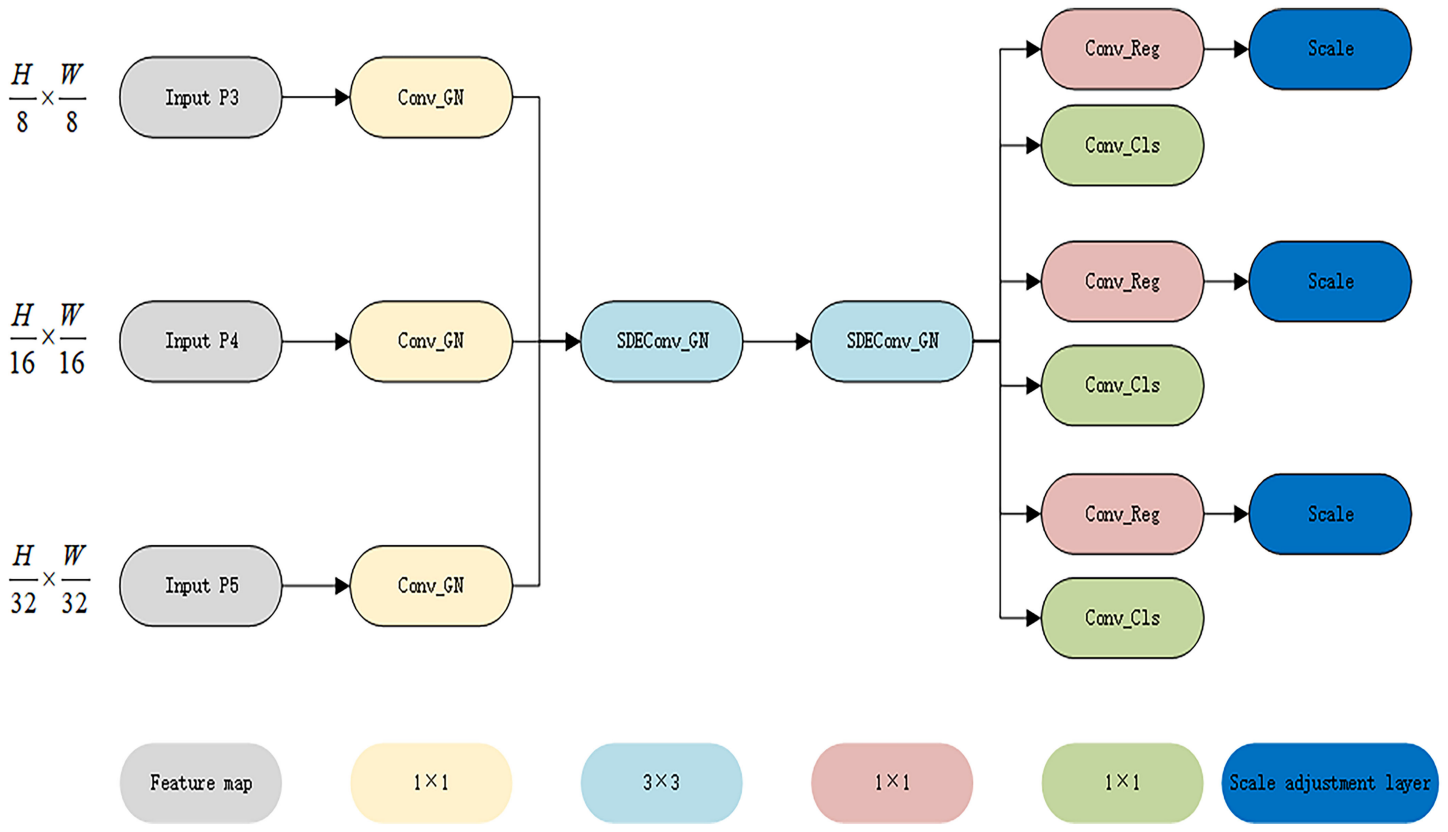
LSDECD structure diagram.

1) To enhance feature extraction capabilities while effectively controlling computational costs hybrid structure called CSP-PTB (Cross Stage Partial–Partially Transformer Block) was designed (**Figure 6**). This module divided the input feature channels into two parts, processed by CNN and Transformer, respectively. CNN extracted local features, while the Transformer obtained global features through multihead self-attention (MHSA), achieving complementarity between local and global information. To avoid greatly increasing computational complexity, only some channels were used for the Transformer block. To further improve nonlinear expression ability, the module introduced an MHSA-CGLU structure, replacing the traditional feed-forward network with a convolutional gated linear unit (CGLU). The structure also supported dynamically adjusting the proportion of channels used for the Transformer according to model size, adapting to different computational resources and task requirements.

2) To improve efficiency and detection of multi-scale targets, the FeaturePyramidSharedConv module was introduced (**Figure 7**). Convolutional layers with different dilation rates captured both local details and global contextual information. Shared convolutional layers (self.share_conv) enabled parameter reuse across feature maps at different scales, ensuring consistent feature representation and efficient fusion. Compared with the pooling operation of SPPF, the convolution operation was more flexible and expressive, capturing fine-grained features and improving multi-scale fusion without significantly increasing model complexity. The sharing mechanism also enhanced the model’s generalization ability, making it more stable when dealing with images that had significant distribution variations or severe interference.

3) YOLO-PFL replaced the traditional YOLO head with an LSDECD (Lightweight Shared Detail Enhanced Convolutional Detection Head) module (Sun et al., 2025). As shown in **Figure 8**, which illustrated the structure of the LSDECD module, a detail-enhanced convolution (DEConv) was designed to integrate prior information into the regular convolution layer, improving representation and generalization capabilities. Through re-parameterization techniques, DEConv was transformed into a regular convolution, eliminating the need for additional parameters and computational costs. This design made the model suitable for deployment on terminal devices with limited computing resources.

#### 2.3.3 3D coordinate acquisition

Stereo vision technology was used for depth estimation. This technique was based on the binocular disparity principle (Yu et al., 2024), where the OAK-D-S2 stereo camera (left camera: CAM_B; right camera: CAM_C) captured the same scene. By matching the same object points in the two images, the positional differences between objects were determined. According to triangulation principles, the relationship between parallax and depth is shown in Equation 1:


(1) $${\text{Z}}_{\text{C}}=\frac{\text{f}\cdot \text{B}}{\text{d}}$$

where *Z*_C_ is the Z-axis distance (depth value) from the target object to the camera in the camera coordinate system, f is the focal length of the camera, B is the baseline length between the two cameras, and d is the disparity, i.e., the horizontal deviation of the corresponding pixels of the same object point in the two images.

This formula was based on triangulation principles, using the camera’s internal parameters and disparity values to calculate the spatial depth of each pixel, thus converting two-dimensional images into spatial position information. To accurately locate objects in 3D space, this study employed a spatial position calculator that combined depth images and camera internal parameters to convert 2D pixels into corresponding 3D coordinates, as shown in Equations 2, 3.


(2) $${\text{X}}_{\text{C}}=\frac{(\text{u}-{\text{c}}_{\text{x}})\cdot {\text{Z}}_{\text{C}}}{{\text{f}}_{\text{x}}}$$

(3) $${\text{Y}}_{\text{C}}=\frac{(\text{v}-{\text{c}}_{\text{y}})\cdot {\text{Z}}_{\text{C}}}{{\text{f}}_{\text{y}}}$$

where (u, v) are the 2D pixel coordinates, c_x_ and c_y_ are the coordinates of the camera’s principal point (usually located at the image center), f_x_ and f_y_ are the horizontal and vertical focal lengths of the camera, respectively, and Z is the depth value.

### 2.4 Estimation of azimuthal of the crop rows in the ridge relative to the camera

#### 2.4.1 Image preprocessing and crop row extraction

**Figure 9:**
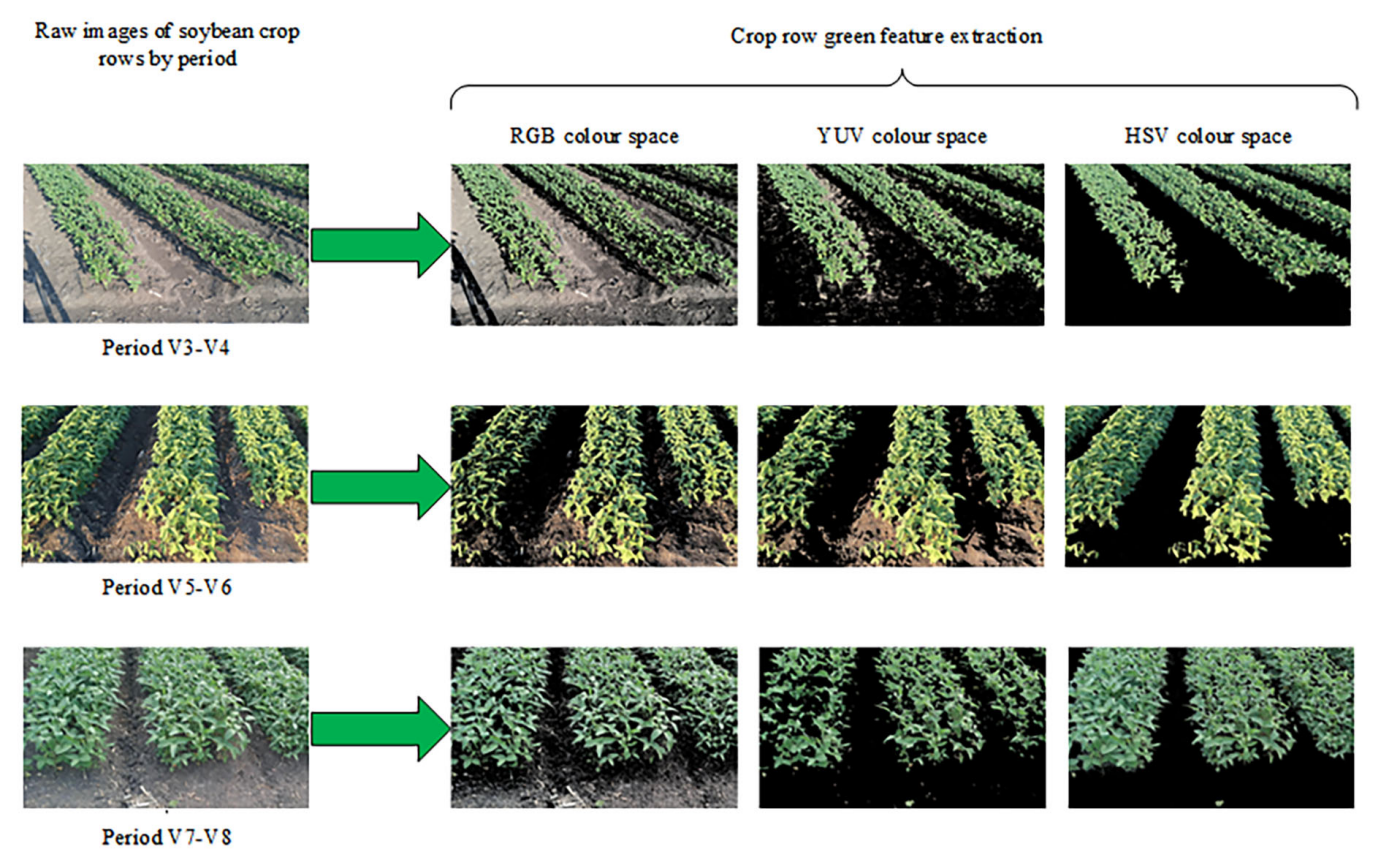
Three color spaces to extract green region comparison images.

The field environment was complex, and background interference factors (such as straw, weeds, and soil), light changes (such as shadows), and crop growth factors (such as missing plants) affected the accuracy of crop row detection and fitting. In the RGB images collected, crop rows usually appeared green as the main color component, and their color difference from the field background was relatively significant. This study used the OpenCV library to convert the collected RGB images into RGB, YUV, and HSV color space models to extract the target green area for comparison. The resulting green threshold mask image is shown in **Figure 9**.

The HSV color space model for crop row green feature extraction more effectively separated soybean crop rows from the background, which led to its selection for image processing. The minimum and maximum HSV values were set according to the hue (H), saturation (S), and value (V) of the green component, calculated as shown in Equations 4, 5:


(4) minGreen=[H_min,S_min,V_min]

(5) maxGreen=[H_max,S_max,V_max]

where H_min is the minimum H-channel value of the green range and H_max is the maximum H-channel value of the green range; S_min is the minimum S-channel value of the green range and S_max is the maximum S-channel value of the green range; V_min is the minimum V-channel value of the green range and V_max is the maximum V-channel value of the green range.

To further process noise and enhance crop row edge features, the mask image was subjected to morphological operations of erosion followed by dilation (Hamuda et al., 2017), as calculated in Equation 6:


(6) (A·B)=(A⊖b)⊕b

where A is the original image, B is the structural element, ⊖ represents the erosion operation, and ⊕ represents the dilation operation.

The erosion convolution kernel was controlled by (6–i, 6–i), and the erosion effect increased with the number of iterations, which helped remove small spurious noise. The dilation convolution kernel was controlled by (i, i), and the dilation effect increased with the number of iterations, which helped connect dispersed green areas. By cycling through three opening operations starting from 1 and ending at 7 with a step size of 2, the resulting mask image was cleaner and more clearly showed the contour of soybean crop rows on the ridge.

Contours were detected using the cv2.findContours() function, and a minimum area threshold was set to filter out small noisy contours. This series of preprocessing and crop row extraction methods effectively separated soybean crop rows from the cluttered field background.

#### 2.4.2 ROI determination and feature point extraction

For accurate extraction of crop row centerline features, multiple ROIs (Zhang et al., 2024) were defined on the image. Each ROI region was a long horizontal strip of pixels with the same width as the image and a height of 40 pixels. The layout of the ROIs was shifted progressively at 60-pixel intervals to ensure full coverage of the crop row area. The main purpose of these ROIs was to delineate the crop rows.

The overlapping areas between ROIs and crop rows were first detected and overlaps that were too small were subsequently removed through area screening to reduce noise. The overlapping portion was identified by intersection detection using a bitwise AND operation, as shown in Equation 7:


(7) Overlap(ROI,Contour)=ROI&Contour

where ROI is the binary image of each ROI region, and Contour is the binary image of the crop row contour.

The center of mass of each mask intersection was then calculated as the extracted feature point, providing the basic data for subsequent straight-line fitting of crop rows and angle calculation. The formulas are shown in Equations 8, 9.


(8) $${\text{C}}_{\text{x}}=\frac{\sum_{\text{x},\text{y}}{\text{x}*\text{C}}_{\left(\text{x},\text{y}\right)}}{\sum_{\text{x},\text{y}}{\text{C}}_{\left(\text{x},\text{y}\right)}}$$

(9) $${\text{C}}_{\text{y}}=\frac{\sum_{\text{x},\text{y}}{\text{y}*\text{C}}_{\left(\text{x},\text{y}\right)}}{\sum_{\text{x},\text{y}}{\text{C}}_{\left(\text{x},\text{y}\right)}}$$

where x and y are the coordinates of each pixel point, C_(x, y)_=1 for pixels in the intersection region and C_(x, y)_=0 for pixels outside the intersection; C_x_ is the center-of-mass coordinate of the intersection region on the x-axis and C_y_ is the center-of-mass coordinate of the intersection region on the y-axis;∑_x, y_ C_(x, y)_ is the total number of pixels in the intersection region.

#### 2.4.3 Crop row centerline fitting and angle calculation

From the set of centers of mass detected within each contour in Section 2.4.1, suppose there are i contours in an image, and each contour contains n centroid points. The set of coordinates of these centroid points is expressed as: C_i_ = {(x_k_, y_k_) | k=1, 2,…, n}. Straight-line fitting was performed using the cv2.fitLine() function, which is based on the least squares criterion of the L2 norm. For a given set of center-of-mass coordinates C_i_, the optimization objective function is calculated as shown in Equation 10.


(10) $$\underset{{\text{v}}_{\text{x}},{\text{v}}_{\text{y}},{\text{x}}_{0},{\text{y}}_{0}}{\min}\overset{\text{n}}{\underset{\text{k}=1}{\sum }}\frac{{\left({\text{v}}_{\text{y}}({\text{x}}_{\text{k}}-{\text{x}}_{0})-{\text{v}}_{\text{y}}({\text{y}}_{\text{k}}-{\text{y}}_{0})\right)}^{2}}{{\text{v}}_{x}^{2}+{\text{v}}_{y}^{2}}$$

By solving this optimization problem, the optimal fitted linear parameters were obtained. The fitted line equation is shown in Equation 11.


(11) {x=x0+vx·t y=y0+vy·t  (t∈R)

The formulas for calculating the endpoints of a straight line based on the image width W are shown in Equations 12, 13.


(12) $${\text{P}}_{\text{start}}=(0,\text{y}{|}_{\text{x}=0})=(0,{\text{y}}_{0}-\frac{{\text{x}}_{0}{\text{v}}_{\text{y}}}{{\text{v}}_{\text{x}}})$$

(13) $${\text{P}}_{\text{end}}=(\text{W},\text{y}{|}_{\text{x}=\text{w}})=(\text{W},{\text{y}}_{0}+\frac{(\text{W}-{\text{x}}_{0}){\text{v}}_{\text{y}}}{{\text{v}}_{\text{x}}})$$

In addition, the angle between the fitted straight line and the horizontal axis was calculated to quantitatively describe the orientation of the crop rows. The slope of the straight line was obtained using the arctangent function and converted into an angle value to determine the orientation of the crop row. This angular information was then used for steering alignment with the crop row, as calculated in Equations 14, 15.


(14) $$\text{β}=\text{arctan}2({\text{v}}_{\text{y}},{\text{v}}_{\text{x}})$$

(15) $${\text{β}}_{\text{deg}}=\frac{180}{\text{π}}\cdot \text{θ}$$

where (x_0_, y_0_) is the reference point of the line, and (v_x_, v_y_) is the direction vector.

### 2.5 Arc steering path generation and visualization in world coordinate system

#### 2.5.1 Circular arc steering path determination

According to Zhou et al. (2022), traditional path planning algorithms encompass graph- search, sampling-based, interpolating curve, and reaction-based algorithms. Among these, the interpolation curve algorithm has a simpler structure and higher computational efficiency than other approaches, making it well suited for real-time systems. Consequently, this method was selected for path planning research in this study.

**Figure 10:**
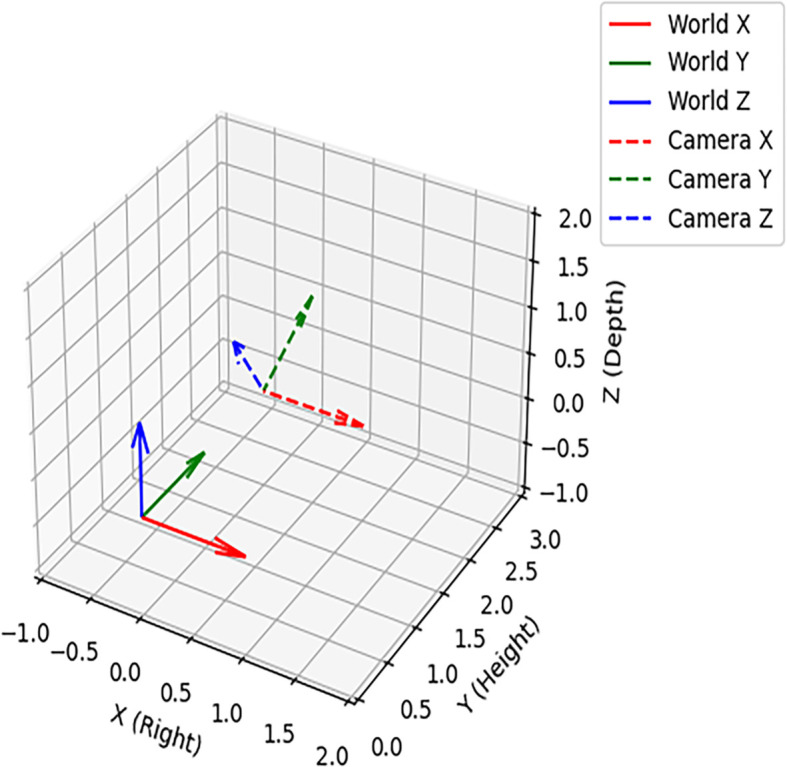
Relationship between the world coordinate system and the camera coordinate system.


The relationship between the world coordinate system and the camera coordinate system as shown in **Figure 10**, the dashed line represented the camera coordinate system (P_C_), the solid line indicated the world coordinate system (P_W_), the world coordinate system World Z (Z_W_) was the forward direction, World Y (Y_W_) was the height perpendicular to the ground, World X (X_W_) was the horizontal direction of the ground, the X_W_ was negative in the right half of the Z_W_ axis and positive in the left half; The camera was rotate around the X_W_ axis by 30.000°, and the origin was set 2 m vertically above the world coordinate system at (0.000, 2.000, 0.000). The translation vector T of the origin of the P_C_ in the P_W_ is shown in Equation 16.

(16) $$\text{T}=[\begin{array}{c} 0\\ 2\\ 0 \end{array}]$$

The transformation from the camera coordinate system to the world coordinate system consisted of two parts: rotation and translation. The rotation matrix of the camera coordinate system around the world coordinate system X_W_ is shown in Equation 17.


(17) $${\text{R}}_{\text{X}}(\text{γ})=[\begin{array}{ccc} 1 & 0 & 0\\ 0 & \cos\text{γ} & -\sin\text{γ}\\ 0 & \sin\text{γ} & \cos\text{γ} \end{array}]$$

γ=30° substituting into Equation 16 gave Equation 18.


(18) $${\text{R}}_{\text{X}}(30{}^{\circ})=[\begin{array}{ccc} 1 & 0 & 0\\ 0 & \frac{\sqrt{3}}{2} & -\frac{1}{2}\\ 0 & \frac{1}{2} & \frac{\sqrt{3}}{2} \end{array}]$$

The position coordinate in the camera coordinate system was [X_C_, Y_C_, Z_C_]^T^ and its corresponding coordinate in the world coordinate system was [X_W_, Y_W_, Z_W_]^T^. The conversion formula is shown in Equation 19.


(19) $${\text{P}}_{\text{W}}={\text{R}}_{X}^{-1}(30{}^{\circ})\cdot {\text{P}}_{\text{C}}+\text{T}$$

The expansion to component form is shown in Equation 20.


(20) $$\left\{ \begin{array}{c} {\text{X}}_{\text{W}}={\text{X}}_{\text{C}}\\ {\text{Y}}_{\text{W}}={\text{Y}}_{\text{C}}\cdot \frac{\sqrt{3}}{2}+{\text{Z}}_{\text{C}}\cdot \frac{1}{2}+2\\ {\text{Z}}_{\text{W}}=-{\text{Y}}_{\text{C}}\cdot \frac{1}{2}+{\text{Z}}_{\text{C}}\cdot \frac{\sqrt{3}}{2}\;\;\;\;\; \end{array}\right.$$

The circular radius of the arc (R) was determined based on the shorter distance of the target in X_W_ and Z_W_ in P_W_. In practical implementation, R is constrained by the vehicle’s minimum turning radius (R_min_). Equation 20 ensures that the computed radius never falls below R_min_. The center of the circular arc (O) was determined based on the X_W_ and Z_W_ values of the target in P_W_ and the radius. The radian (α) was determined by the angle between the crop row centerline and the horizontal axis (β_deg_) calculated in Section 2.4.3, which represents the angle formed between the crop row centerline and the Z_W_ direction. The radius is shown in Equation 21.


(21) $$\text{R}=\text{min}\left[\left|{\text{X}}_{\text{W}}\right|,\left|{\text{Z}}_{\text{W}}\right|\right]\;(\text{R}>{\text{R}}_{\text{min}})$$

The center of the circle is shown in Equations 22-24.


(22) $$\{\begin{array}{c} \text{O}=\left(\text{R},0,{\text{Z}}_{\text{W}}-\text{R}\right)\;\;\;\;\;\;\;\;\;\;(\text{R}=\left|{\text{X}}_{\text{W}}\right|,{\text{X}}_{\text{W}}>0)\\ \text{O}=\left(-\text{R},0,{\text{Z}}_{\text{W}}-\text{R}\right)\;\;\;\;\;(\text{R}=\left|{\text{X}}_{\text{W}}\right|,{\text{X}}_{\text{W}}<0) \end{array}$$

(23) $$\{\begin{array}{c} \text{O}=\left(\text{R},0,0\right)\;\;\;\;\;\;\;\;\;\;\;\;(\text{R}={\text{Z}}_{\text{W}},{\text{X}}_{\text{W}}>0)\\ \text{O}=\left(-\text{R},0,0\right)\;\;\;\;\;\;\;(\text{R}={\text{Z}}_{\text{W}},{\text{X}}_{\text{W}}<0) \end{array}$$

(24) $$\{\begin{array}{c} \text{O}=\left(\text{R},0,0\right)\;\;\;\;\;\;\;\;\;\;\;\;(\text{R}={\text{Z}}_{\text{W}}=\left|{\text{X}}_{\text{W}}\right|,{\text{X}}_{\text{W}}>0)\\ \text{O}=\left(-\text{R},0,0\right)\;\;\;\;\;\;\;(\text{R}={\text{Z}}_{\text{W}}=\left|{\text{X}}_{\text{W}}\right|,{\text{X}}_{\text{W}}<0) \end{array}$$

The radian is shown in Equation 25.


(25) $$\{\begin{array}{c} \alpha ={\text{β}}_{\text{deg}}\;\;\;\;\;\;\;\;\;({\text{β}}_{\text{deg}}>0)\\ \alpha =180^{{}^{\circ}}+{\text{β}}_{\text{deg}}\;\;({\text{β}}_{\text{deg}}<0) \end{array}$$

By determining the three values of R, O, and α, the vehicle’s steering path can be established. To ensure a smooth and continuous trajectory, cubic spline interpolation was employed to transition between the current vehicle heading and the target arc, minimizing abrupt steering changes. Specifically, the cubic spline interpolation function was constructed using the scipy.interpolate.CubicSpline library in Python, which is shown in Equation 26.


(26) $${\text{S}}_{\text{i}}\left(\text{x}\right)={\text{a}}_{\text{i}}+{\text{b}}_{\text{i}}\left(\text{x}-{\text{x}}_{\text{i}}\right)+{\text{c}}_{\text{i}}{\left(\text{x}-{\text{x}}_{\text{i}}\right)}^{2}+{\text{d}}_{\text{i}}{\left(\text{x}-{\text{x}}_{\text{i}}\right)}^{3}\text{ x}\in \left[{\text{x}}_{\text{i}},{\text{x}}_{\text{i}+1}\right]$$

Here, S_i_(x) is the cubic polynomial on the interval [x_i_, x_i+1_], x_i_, x_i+1_ are adjacent data points along the independent variable, a_i_, b_i_, c_i_, d_i_ are coefficients representing the function value and the first-, second-, and third-order terms, respectively; and x is the independent variable, typically representing a path parameter such as distance or time.

By constructing piecewise cubic polynomials that satisfy the continuity of function values as well as first and second derivatives, the vehicle’s heading and path transition smoothly, avoiding sudden steering angle changes and enhancing driving safety.

#### 2.5.2 Path 2D coordinate projection

In the world coordinate system, the circular arc turning path was defined as a series of 3D coordinate points. Each point in the world coordinate system was converted to the camera coordinate system using the camera rotation matrix R and translation vector T. The conversion formulas are shown in Equations 27, 28.


(27) $${\text{P}}_{\text{C}}={\text{R}}_{\text{X}}\left(30{}^{\circ}\right)\cdot \left({\text{P}}_{\text{W}}-\text{T}\right)$$

(28) $$\{\begin{array}{c} {\text{X}}_{\text{C}}={\text{X}}_{\text{W}}\;\\ {\text{Y}}_{\text{C}}=\left({\text{Y}}_{\text{W}}-2\right)\cdot \frac{\sqrt{3}}{2}-{\text{Z}}_{\text{W}}\cdot \frac{1}{2}\\ {\text{Z}}_{\text{C}}=({\text{Y}}_{\text{W}}-2)\cdot \frac{1}{2}+{\text{Z}}_{\text{W}}\cdot \frac{\sqrt{3}}{2} \end{array}$$

The 3D points in the camera coordinate system were then projected onto the 2D image plane using the camera’s intrinsic matrix K. The projection process is given in Equations 29–32:


(29) $${\text{P}}_{2\text{D}}={\left[\text{u},\text{v},1\right]}^{\text{T}}$$

(30) $$\text{K}=\left[\begin{array}{ccc} {\text{f}}_{\text{x}} & 0 & {\text{c}}_{\text{x}}\\ 0 & {\text{f}}_{\text{y}} & {\text{c}}_{\text{y}}\\ 0 & 0 & 1 \end{array}\right]$$

(31) $${\text{P}}_{2\text{D}}={\left[\text{u},\text{v},1\right]}^{\text{T}}=\text{K}\cdot {\left[{\text{X}}_{\text{C}},{\text{Y}}_{\text{C}},{\text{Z}}_{\text{C}}\right]}^{\text{T}}$$

(32) $$\{\begin{array}{c} \text{u}=\frac{{\text{f}}_{\text{x}}\cdot {\text{X}}_{\text{C}}}{{\text{Z}}_{\text{C}}}+{\text{c}}_{\text{x}}\\ \text{v}=\frac{{\text{f}}_{\text{y}}\cdot {\text{Y}}_{\text{C}}}{{\text{Z}}_{\text{C}}}+{\text{c}}_{\text{y}} \end{array}$$

where u and v are the pixel positions in the image coordinate system; K is the intrinsic camera matrix containing parameters such as focal length and principal point; f_x_ and f_y_ are the focal lengths of the camera expressed in pixel units; and c_x_ and c_y_ are the coordinates of the camera’s principal point.

With this projection formula, each point in the camera coordinate system could be mapped to the image coordinate system. Because of camera lens distortion, the points obtained by projecting directly from the camera coordinate system to the image plane might contain errors. Distortion coefficients were therefore applied for correction. The OpenCV undistortPoints function was used to correct the projected points so that they more accurately matched the true position in the image. At the same time, the projection points were shifted and constrained to avoid mapping them beyond the image boundaries, ensuring they always lay within the valid image range.

After completing the conversion, the resulting circular arc path was visualized on the image plane by drawing each projected point. By traversing the points on each arc and marking them in the image using the circle function, the final result was a clearly visualized circular path that provided an accurate navigation reference for the subsequent autopilot system, ensuring that the path planning of the agricultural machine could adapt to changes in the field environment.

## 3 Results and discussion

### 3.1 Crop row headland positioning assessment

#### 3.1.1 Experimental environment and parameters

The experiments were conducted on a computational platform featuring an NVIDIA GeForce RTX 4090 GPU (24 GB VRAM), an Intel Core i9-13900K processor, and 128 GB of system memory under Windows 10 OS. The YOLO architecture was implemented using PyTorch 2.1.2 with Python 3.8.18 and CUDA 12.1 acceleration. A batch size of 16 and input image dimensions of 640 × 640 pixels were configured, and the model was trained for 200 epochs.

#### 3.1.2 Model evaluation criteria

To evaluate the designed model, commonly used performance metrics were applied. These included precision (P), recall (R), mean average precision (mAP@0.5), floating point operations (FLOPs), and number of parameters (Params). Each metric reflected the performance of the detection algorithm from a different perspective. The formulas for these metrics are shown in Equations 33–36.


(33) $$\text{P}=\frac{{\text{T}}_{\text{P}}}{{\text{T}}_{\text{P}}+{\text{F}}_{\text{P}}}$$

(34) $$\text{R}=\frac{{\text{T}}_{\text{P}}}{{\text{T}}_{\text{P}}+{\text{F}}_{\text{N}}}$$

(35) $$\text{AP}=\int_{0}^{1}\text{P}\left(\text{R}\right)\text{dR}$$

(36) $$\text{mAP}=\frac{1}{\text{N}}\sum_{\text{i}=0}^{\text{n}}{\text{AP}}_{\text{i}}$$

where TP is the number of positive samples predicted correctly, FN is the number of negative samples predicted incorrectly, FP is the number of positive samples predicted incorrectly, and N is the total number of all categories.

#### 3.1.3 Comparison of different models and ablation tests

To validate the superiority of the proposed algorithm, comparative experiments were conducted between the improved YOLO-PFL model and RT-DETR, YOLOv6n, and YOLOv8n. The comparison results for each performance index are shown in **Table 2**.

**Table 2:** Comparison of detection results of different models.

Model	P (%)	R (%)	mAP@0.5 (%)	FLOPs (GFLOPs)	Params (M)
RT-DETR	0.900	0.909	0.942	103.433	30.142
YOLOv6n	0.906	0.912	0.937	11.776	4.234
YOLOv8n	0.940	0.900	0.949	8.085	3.006
YOLO-PFL	0.941	0.927	0.956	4.816	1.974

As shown in **Table 2**, compared with other models, the improved YOLO-PFL headland detection model achieved the best mAP@0.5, precision, and recall, reaching 94.100%, 92.700%, and 95.600%, respectively. Its mAP@0.5 was 1.400, 1.900, and 0.700 percentage points higher than RT-DETR, YOLOv6n, and YOLOv8n, respectively, indicating more stable recognition performance. After improvements, the model’s parameters and FLOPs were reduced to 1.974 M and 4.816 GFLOPs, with a significant lightweight effect and the lowest memory footprint among the models tested.

The performance indices of the ablation experiments are shown in **Table 3**. Model “a” represents the baseline YOLOv8n model and serves as a reference for comparing the effects of different module additions. Group B achieved significant optimization by adopting the CSP_PTB module as the backbone structure. Compared with YOLOv8n, Params and FLOPs were reduced by 5.937% and 10.479%, while recall and mAP@0.5 increased by 2.889% and 1.054%. Comparing groups g and h revealed that group g, which did not use the CSP_PTB module, had poorer accuracy and higher Params and FLOPs. Thus, CSP_PTB not only improved model accuracy but also enhanced model lightness. After changing the SPPF to FeaturePyramidSharedConv in group b, recall increased by 0.540%. When YOLOv8n’s SPPF was replaced with FeaturePyramidSharedConv, recall and mAP@0.5 increased by 5.444% and 0.738%, respectively, showing that the FeaturePyramidSharedConv module improved feature extraction ability. Comparing groups a and d showed that using the LSDECD detection head improved recall by 2.667% compared with YOLOv8n, while reducing the Params by 23.886% and FLOPs by 34.508%, with significant reductions in model complexity and parameter size. After replacing the detection head with LSDECD on the basis of group b, FLOPs and Params were reduced by 36.673% and 26.644%, but other evaluation indices decreased. This was attributed to the DEConv detail-enhanced convolution, which substantially reduced FLOPs and Params relative to ordinary convolution. The lightweighting of the model inevitably led to some accuracy loss. The experimental results showed that, compared with other combinations, the improved model proposed in this study had the best overall performance. Precision, recall, and mAP@0.5 reached 94.100%, 92.700%, and 95.600%, respectively. Compared with YOLOv8n, parameters and computation decreased by 40.433% and 34.331%. The significant lightweighting effect confirmed the effectiveness of the improved algorithm.

**Table 3:** Results of ablation experiments.

Model	Model				Index				
	YOLOv8n	CSP-PTB	Feature pyramid shared conv	LSDECD	P (%)	R (%)	mAP@0.5 (%)	FLOPs (GFLOPs)	Params (M)
a	✓				0.940	0.900	0.949	8.085	3.006
b	✓	✓			0.929	0.926	0.959	7.605	2.691
c	✓		✓		0.935	0.949	0.956	8.085	3.006
d	✓			✓	0.924	0.924	0.947	5.295	2.288
e	✓	✓	✓		0.926	0.931	0.951	7.605	2.691
f	✓	✓		✓	0.929	0.911	0.956	4.816	1.974
g	✓		✓	✓	0.935	0.928	0.953	5.295	2.288
h	✓	✓	✓	✓	0.941	0.927	0.956	4.816	1.974

It is worth noting that in certain cases (e.g., models a and c, b and e, d and g, and f and h), Params and FLOPs remained identical despite the addition of extra modules. This phenomenon likely results from shared backbone structures and the implementation of modules such as FeaturePyramidSharedConv and LSDECD, which reuse existing layers via shared weights. Additionally, the YOLOv8 architecture involves implicit layer fusion and model pruning during export or inference, causing some modules to have negligible impact on total parameter count.

#### 3.1.4 Comparative validation of YOLO-PFL model detection

**Figure 11:**
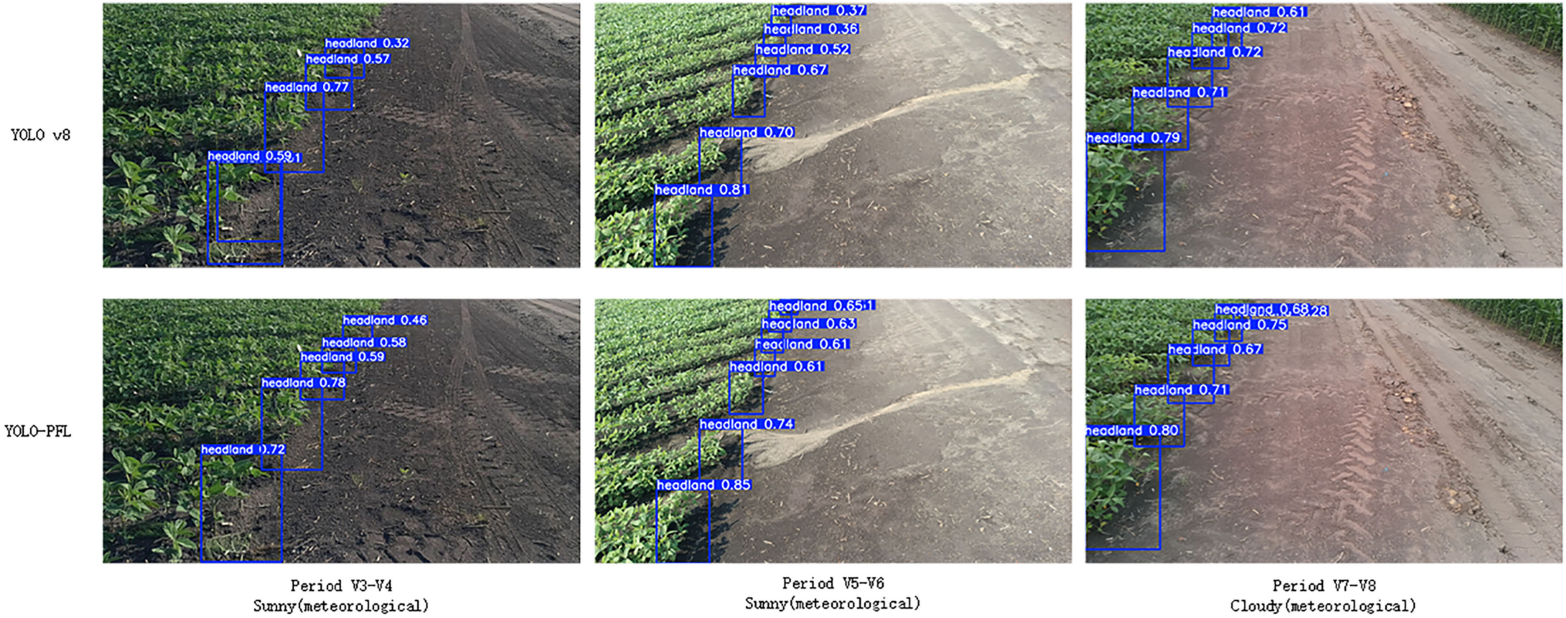
Soybean headland under different models.

To qualitatively evaluate detection performance, the results of the improved YOLOv8 model and YOLOv8n model were compared using images from the test set. Images from the V3–V8 stages under sunny and cloudy conditions were selected, with comparative results shown in **Figure 11**.

As shown in **Figure 11**, the improved YOLOv8n model exhibited no missed detections, false detections, or duplicate detections compared with the other models. In contrast, the original YOLOv8 model had three missed detections and one false detection. The issues in the comparison model were mainly due to incomplete headland images, missing seedlings at the headland, and excessive distance. The improved model accurately identified the headland with high recognition accuracy, demonstrating that the enhanced algorithm could detect targets that were previously undetectable by other models.

It can be seen that the improved YOLO-PFL model has more obvious advantages compared to the YOLOv8n model, which reduces the amount of computation and the number of parameters and saves storage space under the premise of guaranteeing detection accuracy and rate. Moreover, the improved YOLOv8n algorithm improves the problem of inaccurate target positioning and target feature recognition in the ridge, which is suitable for the embedded terminals of agricultural machines.

#### 3.1.5 Three-dimensional spatial positioning accuracy analysis

To verify the accuracy of identifying target positions in 3D space, the positioning accuracy of stereo vision–based 3D coordinates was evaluated. By comparing target space coordinates with actual measurements, the errors (ΔX, ΔY, ΔZ) in the X, Y, and Z directions were calculated for each point. The Euclidean distance error and relative error were further calculated, as shown in Equations 37–39.


(37) $$\begin{array}{c} \{\begin{array}{c} \text{Δ}\text{X}=\left|{\text{X}}_{\text{measured}}-{\text{X}}_{\text{actual}}\right|\\ \text{Δ}\text{Y}=\left|{\text{Y}}_{\text{measured}}-{\text{Y}}_{\text{actual}}\right|\\ \text{Δ}\text{Z}=\left|{\text{Z}}_{\text{measured}}-{\text{Z}}_{\text{actual}}\right| \end{array}\;\; \end{array}$$

(38) $$\text{Euclidean Error}=\sqrt{{\left(\text{Δ}\text{X}\right)}^{2}+{\left(\text{Δ}\text{Y}\right)}^{2}+{\left(\text{Δ}\text{Z}\right)}^{2}}$$

(39) Relative Error=(Euclidean ErrorX  actual2+Y  actual2+Z  actual2×100%)

where X_measured_ is the measured X coordinate value (obtained using the stereo vision method), Y_measured_ is the measured Y coordinate value, and Z_measured_ is the measured Z coordinate value; X_actual_ is the actual X coordinate value (the real position of the target), Y_actual_ is the actual Y coordinate value, and Z_actual_ is the actual Z coordinate value.

The experiment was conducted under ten different distance conditions: Xactual and Yactual were fixed at 1.000 m and 2.000 m respectively, and Zactual ranged from 1.000 to 10.000 m, as shown in **Table 4**. By comparing the actual distances with the 3D coordinates at distances ≤3.000 m, within 4.000% at 3.000–7.000 m, and within 6.000% at 7.000–10.000 m. These results indicate good 3D positioning capability.

**Table 4:** Table of results of 3D positioning error assessment.

Number	True coordinates (m)	Measured coordinates (m)	ΔX (m)	ΔY (m)	ΔZ (m)	Euclidean error (m)	Relative error (%)
1	(1.000, 2.000, 1.000)	(1.010, 1.990, 0.990)	0.010	0.010	0.010	0.017	0.707
2	(1.000, 2.000, 2.000)	(0.990, 2.030, 1.960)	0.010	0.030	0.040	0.051	1.700
3	(1.000, 2.000, 3.000)	(1.020, 2.030, 2.950)	0.020	0.030	0.050	0.062	1.657
4	(1.000, 2.000, 4.000)	(1.020, 2.040, 4.150)	0.020	0.040	0.150	0.157	3.424
5	(1.000, 2.000, 5.000)	(0.960, 2.070, 5.180)	0.040	0.070	0.180	0.197	3.597
6	(1.000, 2.000, 6.000)	(1.030, 1.920, 5.780)	0.030	0.080	0.220	0.236	3.686
7	(1.000, 2.000, 7.000)	(1.040, 1.930, 7.260)	0.040	0.070	0.260	0.272	3.702
8	(1.000, 2.000, 8.000)	(0.970, 2.080, 7.650)	0.030	0.080	0.350	0.360	4.333
9	(1.000, 2.000, 9.000)	(1.060, 2.110, 9.350)	0.060	0.110	0.350	0.372	4.011
10	(1.000, 2.000, 10.000)	(0.950, 2.120, 9.430)	0.050	0.120	0.570	0.585	5.709

### 3.2 Evaluation of the accuracy of directional angle fitting for crop rows in ridges

#### 3.2.1 Analysis of the effect of image preprocessing on crop row structure recognition

**Figure 12:**
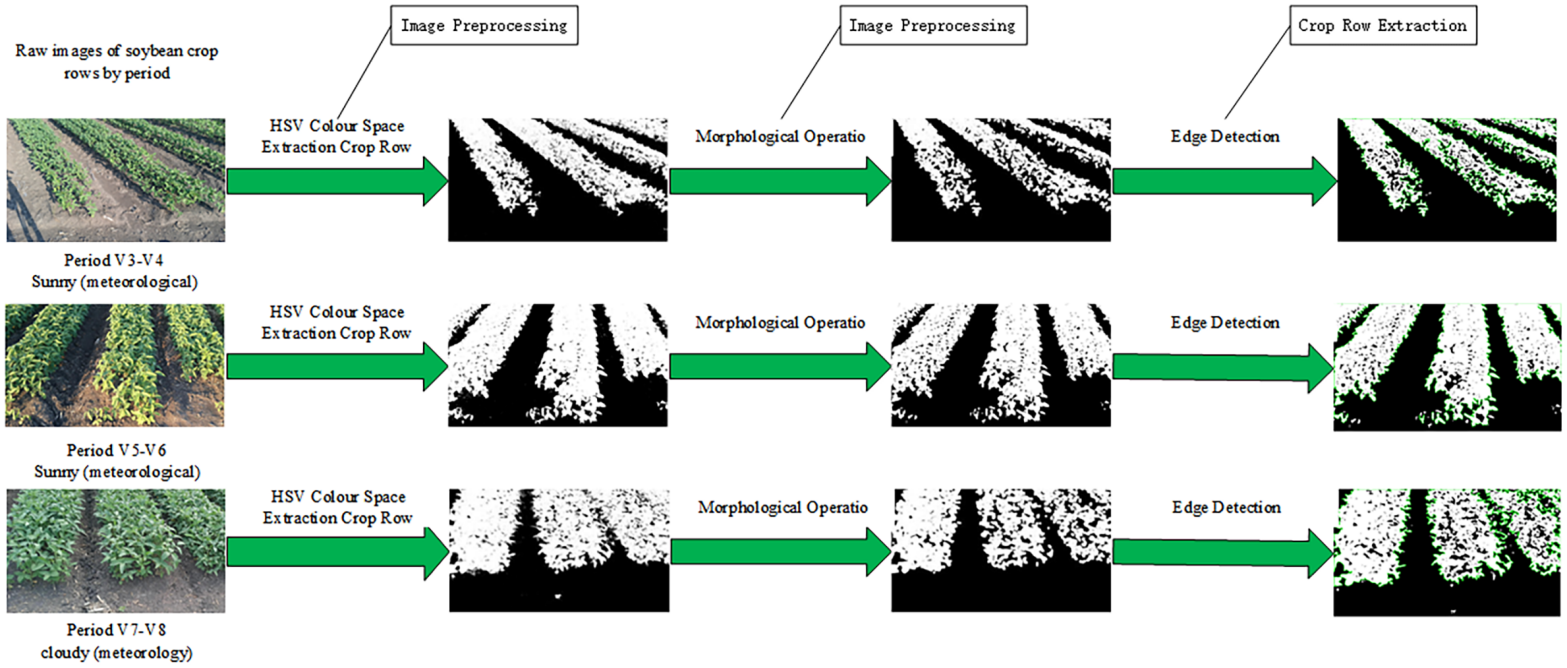
Image preprocessing and crop row extraction results.

The results of image preprocessing and crop row extraction on the original images are shown in **Figure 12**. It was observed that straw interference appeared in these images, and shadows were produced under sunny conditions. By extracting the green channel component and generating the corresponding binarized image through the HSV color space model, non-green background information—such as straw, soil, and shaded areas—was effectively removed. As a result, the crop area and the background area could be clearly separated. This finding verifies the robustness of the HSV color space in extracting green crop areas under complex lighting and background interference conditions.

During the V7–V8 growth stage of soybean plants, the crop rows between two ridges exhibited some adhesion, and holes appeared within the same ridge. After applying morphological operations, small noise was eliminated, and the boundaries of larger objects were smoothed to reduce row adhesion. At the same time, the holes in the mask were filled, ensuring that the object area was more continuous and complete. This indicates that the preprocessing method used in this study demonstrates strong adaptability and stability in complex field environments, providing a solid foundation for subsequent contour extraction of crop rows.

The soybean crop rows were completely recognized after contour extraction, with clear contour boundaries. Their alignment direction was consistent with the actual planting direction, verifying the effectiveness of the method. In particular, in this regular planting structure, the extraction results were highly consistent with the actual situation, indicating that the method has strong adaptability for structured planting scenarios.

#### 3.2.2 Feature point extraction

**Figure 13:**
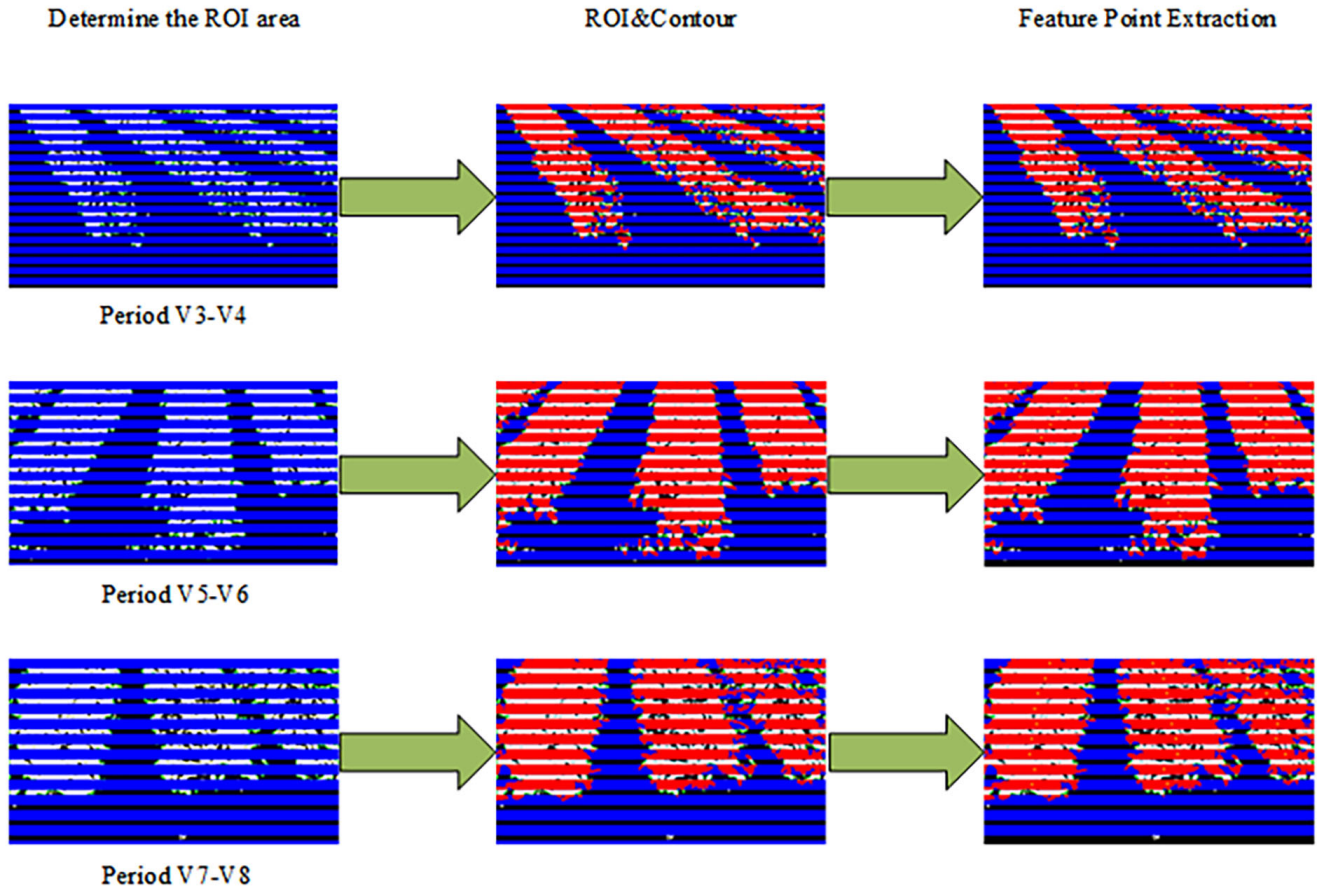
Results of the feature point extraction process.

As shown in **Figure 13**, multiple ROIs were constructed on the image from which the crop row contours were extracted. These were divided into a series of blue-banded regions from top to bottom, effectively enhancing the local structural analysis of longitudinal crop row distribution. The intersection portions between the ROI regions and the closed contours of the crop rows were marked in red, visualizing the spatial distribution of the crop rows in each ROI. This operation “cut” a continuous crop row into multiple smaller regions with distinct boundaries, which helped capture the local morphological features of the crop row.

Further, by extracting the center of mass of these intersecting regions and marking them in orange, the method provided stable and representative geometric reference points for subsequent crop row centerline extraction. However, some holes or fractures appeared in certain intersection regions, leading to an abnormal distribution of center-of-mass points. To address this problem, an area-based screening strategy was introduced to remove small regions with areas below a set threshold, thus effectively reducing the noise interference. Experimental results showed that this strategy significantly improved the spatial stability and distribution continuity of the center-of-mass points. Overall, the proposed lateral ROI division and intersection center-of-mass extraction method lays a good foundation for the subsequent crop row centerline identification task.

#### 3.2.3 Centerline fitting results and direction angle accuracy assessment

**Figure 14:**
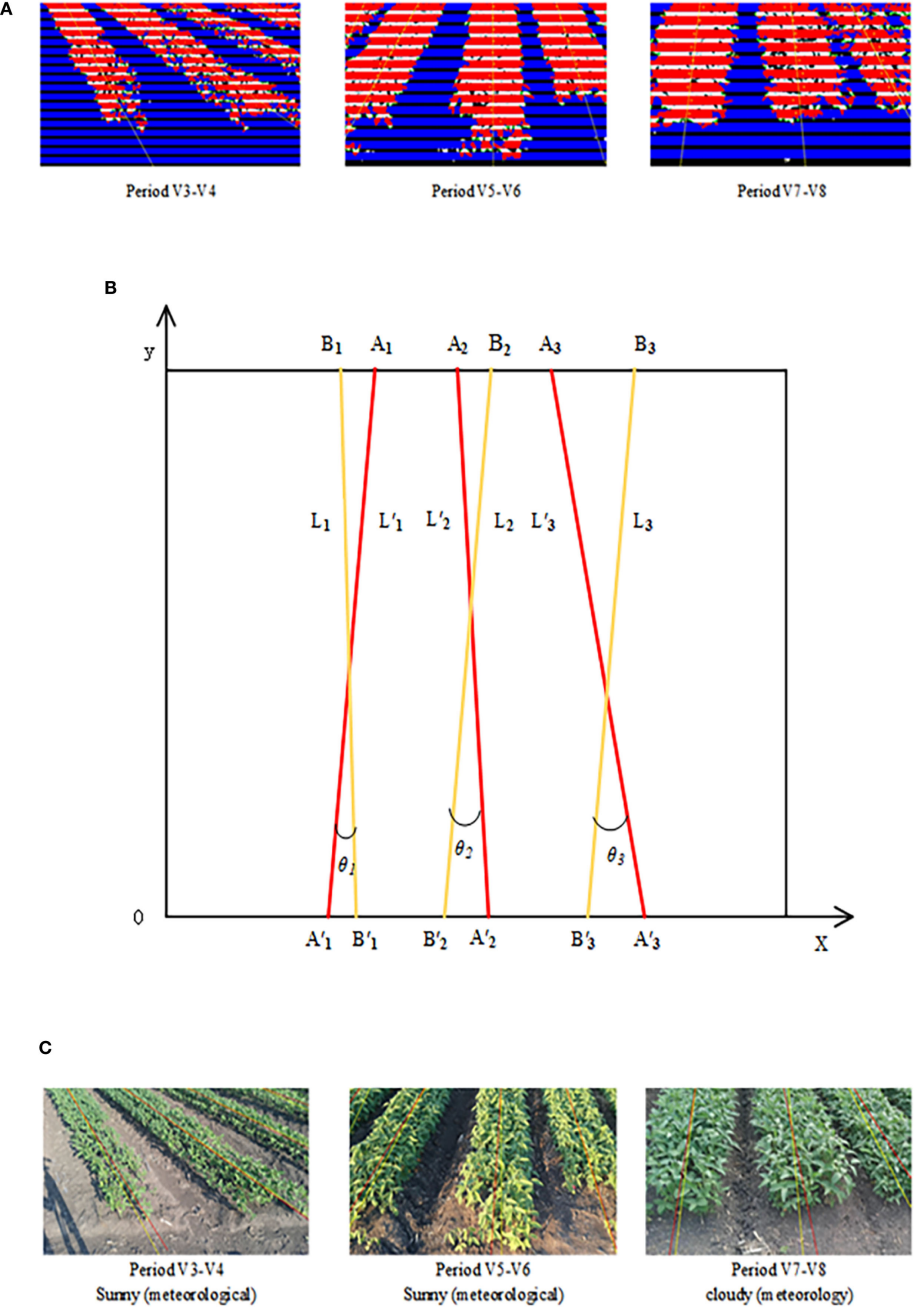
Angle accuracy assessment. **(A)** Crop row centerline fitting results. **(B)** Schematic diagram of the accuracy verification method. **(C)** Comparison results of the standard line with the fitted straight line.

The centers of mass within the contour of the crop row were fitted using the least squares method to obtain the crop row centerline, as shown in **Figure 14A**. To assess the accuracy of the fitting, a standard line was constructed by joining the midpoints of the top and bottom edges of the crop rows and extending them. This standard line was used as the reference for evaluating the fitted centerline. To make the verification results more rigorous, accuracy was verified by calculating the angular difference between the fitted crop row centerline and the standard line.

The verification principle of accuracy is shown in **Figure 14B**, L′_1_, L′_2_ and L′_3_ represent the standard lines, while L_1_, L_2,_ and L_3_ are the lines fitted through feature points. A_1_, A_2_, A_3_, A′_1_, A′_2_ and A′_3_ are the endpoint coordinates of the three standard lines, and B_1_, B_2_, B_3_, B′_1_, B′_2_ and B′_3_ are the endpoint coordinates of the three fitted lines. The angle θ between the fitted line and the standard line may be either positive or negative, reflecting both the deviation angle and the deviation direction. A negative θ indicates that the fitted line deviates to the left of the standard line, while a positive θ indicates that it deviates to the right. θ_1_ is the deviation angle between L_1_ and L′_1_, indicating their relative position; absθ_1_ is the absolute value of θ_1_, indicating the magnitude of angular deviation.

Let the two endpoints of the standard line be (x_1_, y_1_) and (x_2_, y_2_), and the two endpoints of the fitted line be (x′_1_, y′_1_) and (x′_2_, y′_2_). The slopes m_1_ and m_2_ of the fitted and standard lines are calculated as shown in Equations 40, 41.


(40) $${\text{m}}_{1}=\frac{{\text{y}}_{2}-{\text{y}}_{1}}{{\text{x}}_{2}-{\text{x}}_{1}}$$

(41) $${\text{m}}_{2}=\frac{{\text{y}\text{'}}_{2}-{\text{y}\text{'}}_{1}}{{\text{x}\text{'}}_{2}-{\text{x'}}_{1}}$$

The equation for the angle θ_1_ was obtained from the slopes of the standard and fitted straight lines, as shown in Equation 42.


(42) $${\text{θ}}_{1}=\text{arctan}\left(\left|\frac{{\text{m}}_{2}-{\text{m}}_{1}}{1+{\text{m}}_{2}{\text{m}}_{1}}\right|\right)$$

The yellow line represents the crop row fitting results, and the red line represents the standard line, as shown in **Figure 14C**. The figure contains three images representing the V3–V4, V5–V6, and V7–V8 periods, respectively. Each image displays k sets of lines and their deviation anglesθ_k_ (k=1,2, onel from left to right. By calculating θ_k_ across the three images from different periods, three sets of angular deviation results (a, b, and c) were obtained, as shown in **Table 5**. It can be seen that the method proposed in this paper maintained high accuracy for crop rows within the ridge during the V3–V8 period, even under conditions such as clear weather, cloudy weather, and excessive straw. The accuracy of the fitting fluctuated when there were missing plants or incomplete crop rows in the image. The average error in the centerline inclination derived from this method was controlled within –0.473°, which was smaller than the average angular deviation of 0.59° obtained by the crop row centerline detection method based on deep convolutional networks proposed by Gong et al. (2024). The maximum error did not exceed 7.000°, and the mean absolute error (MAE) was 3.309°. These results indicate that the accuracy of the method in fitting a straight line was high, meeting the requirements for fitting crop row direction angles within the ridge.

**Table 5:** Sample of angular deviation results (degrees).

Growth Stage	θ_1_	θ_2_	θ_3_	θ_4_
V3–V4 (a)	-3.900°	0.900°	1.000°	-5.000°
V5–V6 (b)	4.800°	3.000°	3.900°	0.100°
V7–V8 (c)	1.900°	-7.000°	-4.900°	

The spatial orientation of the crop rows was described by calculating the angle between the centerline and the horizontal axis of the camera image. Expressing the inclination angle between the fitted line and the horizontal axis in angular form made the results more intuitive and clear. The resulting inclination angles were reasonably distributed among different image regions, and the change trends were consistent with the crop sowing direction. This demonstrates that the centerline fitting method has good accuracy and stability in describing crop row orientation, providing reliable angular information for subsequent applications such as crop row direction estimation and automatic navigation of agricultural machinery.

### 3.3 Steering path planning results and image space visualization

To achieve autonomous steering navigation for wheeled agricultural machines, this study dynamically determined the radius, radian, and center position of the arc based on the positioning of soybean crop rows at the headland and the relative azimuth angle between the rows and the camera. A circular arc model was constructed to simulate the steering path. The path was presented in image space, requiring the transformation of arc path points from the world coordinate system to the camera coordinate system through coordinate transformation. Then, using the intrinsic matrix of CAM_B, the 3D points were projected onto the image plane captured by CAM_B. The projection results were subsequently processed with distortion correction and image boundary constraints.

**Figure 15:**
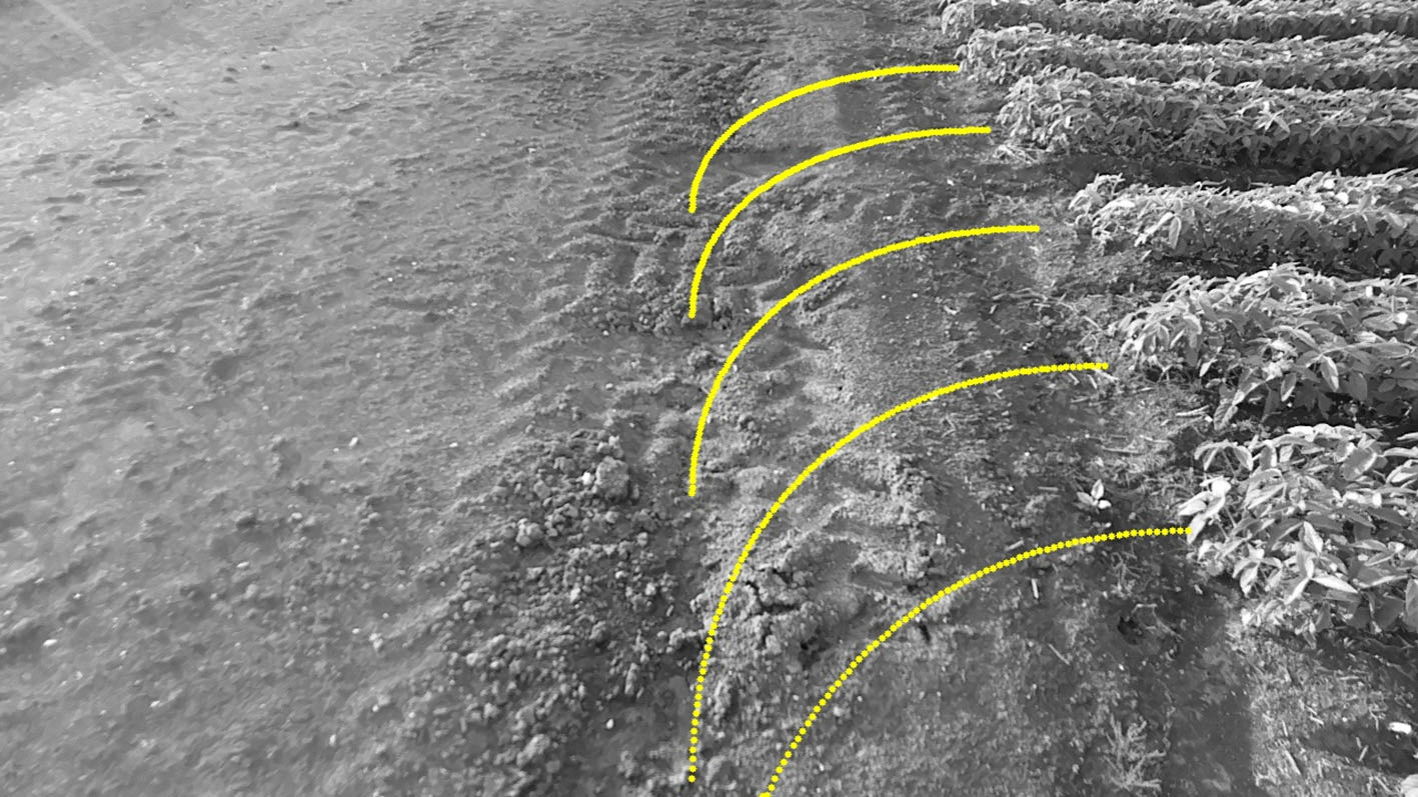
Path visualization results.

The path visualization results in the image space are shown in **Figure 15**. The circular arc path appears as a continuous line in the image, with a well-distributed layout and a smooth transition in direction, without jumps or distortion. Visualization of the path in the image plane provides a clear guidance trajectory for the navigation system of the wheeled agricultural machine, supporting the subsequent design of path tracking and control algorithms. This provides a solid foundation and visualization support for the construction of a robust and efficient field autopilot system.

## 4 Conclusions

This study proposed a method for visualizing headland turning navigation lines in soybean fields. By combining a deep learning–based object localization method with feature detection algorithms, the system precisely detected headlands and obtained their relative positions (the improved model achieved a precision of 94.100%, recall of 92.700%, and mAP@0.5 of 95.600%, with Params and FLOPs of 1.974 M and 4.816 GFLOPs, respectively; distance error was within 2.000% at ≤3.000 m, within 4.000% at 3.000–7.000 m, and within 6.000% at 7.000–10.000 m). The system also successfully extracted crop row centerlines (average error –0.473°, indicating a slight systematic leftward bias; maximum error ≤7.000°; MAE 3.309°). Based on these data, efficient steering path planning and visualization were achieved through image plane projection. Experimental results confirmed high detection accuracy, robust 3D positioning precision, and stable path planning performance, meeting the practical requirements for autonomous driving of wheeled agricultural machinery during the V3–V8 growth stages of soybean fields.

Nonetheless, some limitations remain. The current experiments lacked comprehensive validation under more challenging scenarios, such as backlighting, high weed density, and varying soil moisture levels. Future work will expand testing conditions to enhance system generalization in complex field environments and explore more efficient feature fusion networks and optimized loss functions to further improve detection accuracy and computational efficiency.

Overall, the proposed method provides a solid theoretical foundation and technical support for navigation path planning in agricultural autonomous driving, offering promising potential for practical application and wider adoption.

## Data Availability

The raw data supporting the conclusions of this article will be made available by the authors, without undue reservation.

## References

[B1] Bai Y. Zhang B. Xu N. Zhou J. Shi J. Diao Z. (2023). Vision-based navigation and guidance for agricultural autonomous vehicles and robots: a review. Comput. Electron. Agric. 205, 107584. doi: 10.1016/j.compag.2022.107584

[B2] Chen Z. Dou H. Gao Y. Zhai C. Wang X. Zou W. (2025). Research on an orchard row centreline multipoint autonomous navigation method based on LiDAR. Artif. Intell. Agric. 15, 221–231. doi: 10.1016/j.aiia.2024.12.003

[B3] Diao Z. Guo P. Zhang B. Zhang D. Yan J. He Z.. (2023). Maize crop row recognition algorithm based on improved UNet network. Comput. Electron. Agric. 210, 107940. doi: 10.1016/j.compag.2023.107940

[B4] Ding H. Zhang B. Zhou J. Yan Y. Tian G. Gu B. (2022). Recent developments and applications of simultaneous localization and mapping in agriculture. J. Field Robot. 39, 956–983. doi: 10.1002/rob.22077

[B5] Gong H. Zhuang W. Wang X. (2024). Improving the maize crop row navigation line recognition method of YOLOX. Front. Plant Sci. 15. doi: 10.3389/fpls.2024.1338228, PMID: 38606066 PMC11008721

[B6] Guo Z. Quan L. Sun D. Lou Z. Geng Y. Chen T.. (2024). Efficient crop row detection using transformer-based parameter prediction. Biosyst. Eng. 246, 13–25. doi: 10.1016/j.biosystemseng.2024.07.016

[B7] Hamuda E. Mc Ginley B. Glavin M. Jones E. (2017). Automatic crop detection under field conditions using the HSV colour space and morphological operations. Comput. Electron. Agric. 133, 97–107. doi: 10.1016/j.compag.2016.11.021

[B8] He Y. Zhang X. Zhang Z. Fang H. (2022). Automated detection of boundary line in paddy field using MobileV2-UNet and RANSAC. Comput. Electron. Agric. 194, 106697. doi: 10.1016/j.compag.2022.106697

[B9] Hong Z. Li Y. Lin H. Gong L. Liu C. (2022). Field boundary distance detection method in early stage of planting based on binocular vision. Trans. Chin. Soc Agric. Mach. 53, 27–33. doi: 10.6041/j.issn.1000-1298.2022.05.003

[B10] Huang Y. Fu J. Xu S. Han T. Liu Y. (2022). Research on integrated navigation system of agricultural machinery based on RTK-BDS/INS. Agric 12, 1169. doi: 10.3390/agriculture12081169

[B11] Li C. Pan Y. Li D. Fan J. Li B. Zhao Y.. (2024). A curved path extraction method using RGB-D multimodal data for single-edge guided navigation in irregularly shaped fields. Expert Syst. Appl. 255, 124586. doi: 10.1016/j.eswa.2024.124586

[B12] Li D. Li B. Long S. Feng H. Wang Y. Wang J. (2023). Robust detection of headland boundary in paddy fields from continuous RGB-D images using hybrid deep neural networks. Comput. Electron. Agric. 207, 107713. doi: 10.1016/j.compag.2023.107713

[B13] Li J. Gao W. Wu Y. Liu Y. Shen Y. (2022). High-quality indoor scene 3D reconstruction with RGB-D cameras: a brief review. Comput. Vis. Media. 8, 369–393. doi: 10.1007/s41095-021-0250-8

[B14] Li S. Zheng C. Wen C. Li K. (2024). Method for locating missing ratoon sugarcane seedlings based on RGB images from unmanned aerial vehicles and improve YOLO v5s. Trans. Chin. Soc Agric. Mach. 55, 57–70. doi: 10.6041/j.issn.1000-1298.2024.12.005

[B15] Li X. Lloyd R. Ward S. Cox J. Coutts S. Fox C. (2022). Robotic crop row tracking around weeds using cereal-specific features. Comput. Electron. Agric. 197, 106941. doi: 10.1016/j.compag.2022.106941

[B16] Lin Y. Xia S. Wang L. Qiao B. Han H. Wang L.. (2025). Multi-task deep convolutional neural network for weed detection and navigation path extraction. Comput. Electron. Agric. 229, 109776. doi: 10.1016/j.compag.2024.109776

[B17] Luo Y. Wei L. Xu L. Zhang Q. Liu J. Cai Q.. (2022). Stereo-vision-based multi-crop harvesting edge detection for precise automatic steering of combine harvester. Biosyst. Eng. 215, 115–128. doi: 10.1016/j.biosystemseng.2021.12.016

[B18] Ma Z. Tao Z. Du X. Yu Y. Wu C. (2021). Automatic detection of crop root rows in paddy fields based on straight-line clustering algorithm and supervised learning method. Biosyst. Eng. 211, 63–76. doi: 10.1016/j.biosystemseng.2021.08.030

[B19] Pin J. Guo T. Xv X. Zou X. Hu W. (2025). Fast extraction of navigation line and crop position based on LiDAR for cabbage crops. Artif. Intell. Agric. 15, 686–695. doi: 10.1016/j.aiia.2025.03.007

[B20] Sun T. Hong Z. Song C. Xiao P. (2025). Lightweight insulator defect detection based on multiscale feature fusion. Laser Optoelectron. Prog. 62, 136–147. doi: 10.3788/LOP241864

[B21] Wang S. Yu S. Zhang W. Wang X. (2023). The identification of straight-curved rice seedling rows for automatic row avoidance and weeding system. Biosyst. Eng. 233, 47–62. doi: 10.1016/j.biosystemseng.2023.07.003

[B22] Wang Z. Zou Y. Lv J. Cao Y. Yu H. (2024). Lightweight self-supervised monocular depth estimation through CNN and transformer integration. IEEE Access 12, 167934–167943. doi: 10.1109/ACCESS.2024.3494872

[B23] Wu T. Guo H. Zhou W. Gao G. Wang X. Yang C. (2025). Navigation path extraction for farmland headlands via red-green-blue and depth multimodal fusion based on an improved DeepLabv3+ model. Eng. Appl. Artif. Intell. 151, 110681. doi: 10.1016/j.engappai.2025.110681

[B24] Xu B. Chai L. Zhang C. (2023). Research and application on corn crop identification and positioning method based on Machine vision. Inf. Process. Agric. 10, 106–113. doi: 10.1016/j.inpa.2021.07.004

[B25] Yang Y. Zhou Y. Yue X. Zhang G. Wen X. Ma B.. (2023). Real-time detection of crop rows in maize fields based on autonomous extraction of ROI. Expert Syst. Appl. 213, 118826. doi: 10.1016/j.eswa.2022.118826

[B26] Yu B. Liu W. Zhang Y. Ma D. Jia Z. Yue Y.. (2024). Accuracy evaluation for *in-situ* machining reference points binocular measurement based on credibility probability. Chin. J. Aeronaut. 37, 472–486. doi: 10.1016/j.cja.2023.04.007

[B27] Zhai Z. Xiong K. Wang L. Du Y. Zhu Z. Mao E. (2022). Crop row detection and tracking based on binocular vision and adaptive Kalman filter. Trans. Chin. Soc Agric. Eng. 38, 143–151. doi: 10.11975/j.issn.1002-6819.2022.08.017

[B28] Zhang Z. Cao R. Peng C. Liu R. Sun Y. Zhang M.. (2020). Cut-edge detection method for rice harvesting based on machine vision. Agronomy 10, 590. doi: 10.3390/agronomy10040590

[B29] Zhang Y. Lai Z. Wang H. Jiang F. Wang L. (2024). Autonomous navigation using machine vision and self-designed fiducial marker in a commercial chicken farming house. Comput. Electron. Agric. 224, 109179. doi: 10.1016/j.compag.2024.109179

[B30] Zhang S. Liu Y. Xiong K. Zhai Z. Zhu Z. Du Y. (2023). Center line detection of field crop rows based on feature engineering. Trans. Chin. Soc Agric. Mach. 54, 18–26. doi: 10.6041/j.issn.1000-1298.2023.S1.003

[B31] Zhang Z. Zhang X. Cao R. Zhang M. Li H. Yin Y.. (2022). Cut-edge detection method for wheat harvesting based on stereo vision. Comput. Electron. Agric. 197, 106910. doi: 10.1016/j.compag.2022.106910

[B32] Zheng Z. Hu Y. Li X. Huang Y. (2023). Autonomous navigation method of jujube catch-and-shake harvesting robot based on convolutional neural networks. Comput. Electron. Agric. 215, 108469. doi: 10.1016/j.compag.2023.108469

[B33] Zheng X. Zheng J. Wang X. Qu F. Jiang T. Tao Z.. (2025). An adaptive recognition method for crop row orientation in dry land by combining morphological and texture features. Soil Tillage Res. 252, 106576. doi: 10.1016/j.still.2025.106576

[B34] Zhou C. Huang B. Fränti P. (2022). A review of motion planning algorithms for intelligent robots. J. Intell. Manuf. 33, 387–424. doi: 10.1007/s10845-021-01867-z

